# Deletion of myeloid HDAC3 promotes efferocytosis to ameliorate retinal ischemic injury

**DOI:** 10.1186/s12974-024-03159-8

**Published:** 2024-07-12

**Authors:** Rami A. Shahror, Esraa Shosha, Carol Morris, Melissa Wild, Shengyu Mu, Gabor Csanyi, Marjan Boerma, Nancy J. Rusch, Abdelrahman Y. Fouda

**Affiliations:** 1 Department of Pharmacology and Toxicology College of Medicine, University of Arkansas for Medical Sciences (UAMS), 4301 West Markham Street, Slot 611, BIOMED-1, B306, Little Rock, Office, AR 72205 USA; 2 Department of Pharmaceutical Sciences, College of Pharmacy, University of Arkansas for Medical Sciences, Little Rock, AR USA; 3 Department of Pharmacology and Vascular Biology Center, Augusta University, Augusta, GA USA; 4 Clinical Pharmacy Department, Cairo University, Cairo, Egypt

**Keywords:** Retinal ischemia-reperfusion injury, Histone deacetylase 3, Microglia, Macrophages, Efferocytosis

## Abstract

Ischemia-induced retinopathy is a hallmark finding of common visual disorders including diabetic retinopathy (DR) and central retinal artery and vein occlusions. Treatments for ischemic retinopathies fail to improve clinical outcomes and the design of new therapies will depend on understanding the underlying disease mechanisms. Histone deacetylases (HDACs) are an enzyme class that removes acetyl groups from histone and non-histone proteins, thereby regulating gene expression and protein function. HDACs have been implicated in retinal neurovascular injury in preclinical studies in which nonspecific HDAC inhibitors mitigated retinal injury. Histone deacetylase 3 (HDAC3) is a class I histone deacetylase isoform that plays a central role in the macrophage inflammatory response. We recently reported that myeloid cells upregulate HDAC3 in a mouse model of retinal ischemia-reperfusion (IR) injury. However, whether this cellular event is an essential contributor to retinal IR injury is unknown. In this study, we explored the role of myeloid HDAC3 in ischemia-induced retinal neurovascular injury by subjecting myeloid-specific HDAC3 knockout (M-HDAC3 KO) and floxed control mice to retinal IR. The M-HDAC3 KO mice were protected from retinal IR injury as shown by the preservation of inner retinal neurons, vascular integrity, and retinal thickness. Electroretinography confirmed that this neurovascular protection translated to improved retinal function. The retinas of M-HDAC3 KO mice also showed less proliferation and infiltration of myeloid cells after injury. Interestingly, myeloid cells lacking HDAC3 more avidly engulfed apoptotic cells in vitro and after retinal IR injury in vivo compared to wild-type myeloid cells, suggesting that HDAC3 hinders the reparative phagocytosis of dead cells, a process known as efferocytosis. Further mechanistic studies indicated that although HDAC3 KO macrophages upregulate the reparative enzyme arginase 1 (A1) that enhances efferocytosis, the inhibitory effect of HDAC3 on efferocytosis is not solely dependent on A1. Finally, treatment of wild-type mice with the HDAC3 inhibitor RGFP966 ameliorated the retinal neurodegeneration and thinning caused by IR injury. Collectively, our data show that HDAC3 deletion enhances macrophage-mediated efferocytosis and protects against retinal IR injury, suggesting that inhibiting myeloid HDAC3 holds promise as a novel therapeutic strategy for preserving retinal integrity after ischemic insult.

**Supplementary Information:**

The online version contains supplementary material available at 10.1186/s12974-024-03159-8.

## Introduction

Individuals across the lifespan suffer from visual impairment and blindness caused by diseases of retinal ischemia that include retinopathy of prematurity in neonates, diabetic retinopathy (DR), and central retinal artery or vein occlusion in older adults. These conditions are collectively termed ischemic retinopathy, and the absence of effective therapies represents a major clinical challenge for efforts seeking to preserve vision and quality of life. The mechanisms underlying the neurovascular injury that is a feature of ischemic retinopathy are poorly understood and this knowledge gap limits the design of new and improved therapeutic interventions. The collective efforts of the scientific community to identify the pathogenic mechanisms that contribute to ischemia-induced neurovascular injury of the retina often rely on animal models including the ischemia-reperfusion (IR) mouse model of retinopathy [1–4].

Myeloid cells such as microglia and macrophages (MΦ) play a critical role in the progression of retinal IR injury [5–9]. Activation and proliferation of microglia and resident MΦ coupled with infiltration of blood-borne monocytes can promote either retinal protection or degeneration depending on the molecular signature and activation state of the myeloid cells [10–12]. We and others have shown that myeloid cells can play a protective role by shifting from a pro-inflammatory phenotype to a reparative phenotype; the latter myeloid cell phenotype contributes to the resolution of retinal injury [6, 7, 10]. Our studies using a mouse model of retinal IR injury showed that systemic MΦ depletion worsens neurodegeneration and increases retinal hemorrhage suggesting that MΦ confer neurovascular protection [6]. However, the underlying mechanisms by which myeloid cells can promote neurovascular repair are poorly understood.

Efferocytosis is the engulfment of apoptotic cells and cell debris by myeloid cells after injury. It is recognized as a reparative event in several pathological conditions including stroke [13, 14], cancer [15, 16], and atherosclerosis [17]. Conversely, impaired efferocytosis can lead to the accumulation of apoptotic cells and damage-associated molecular patterns (DAMPs) after injury, which fuels a feed-forward cycle of inflammation and further cell death. Reports of efferocytosis in the injured retina have focused primarily on photoreceptor degeneration and conflicting results have documented both reparative and deleterious roles of myeloid cell-mediated phagocytosis [18–21]. Microglia/MΦ-mediated phagocytosis in the inner retina has been documented after retinal IR injury, optic nerve crush, and other ocular injuries, but knowledge of its mechanistic basis and impact on injury progression is scarce [10, 22–24].

Histone deacetylases (HDACs) are a family of enzymes that remove acetyl groups from histone and non-histone proteins and thereby enable chromatin remodeling to regulate gene expression. The HDACs regulate several cellular processes including cell metabolism, proliferation, and the immune response [25]. HDAC inhibitors are reported to be neuroprotective, implying that HDACs perpetrate neuronal injury. For example, general inhibitors of Class I and II histone deacetylases (HDACs) limit neuronal damage in the murine model of retinal IR injury [26, 27], suggesting that specific HDAC isoforms within these two HDAC classes may participate in the underlying pathogenesis. In this regard, recent attention has been drawn to the HDAC3 isoform belonging to the Class I HDACs as a potential culprit. Neuronally expressed HDAC3 has been implicated in ganglion cell degeneration caused by optic nerve crush [28, 29]. HDAC3 also is reported to mediate the epigenetic modifications that promote the expression of MΦ inflammatory genes [30, 31]. We recently reported an upregulation of HDAC3 in myeloid cells in a mouse model of retinal IR injury, suggesting a strong presence of this specific HDAC isoform at the affected site. We additionally discovered that arginase 1 (A1), a reparative enzyme that hydrolyzes arginine and promotes MΦ-mediated efferocytosis, and resolution of retinal IR injury, suppresses HDAC3 [5, 13, 32, 33]. These findings provide indirect evidence to implicate myeloid HDAC3 in retinal IR injury and emphasize the importance of defining its precise role in retinal injury progression and loss of function.

Here, we present data showing for the first time that deletion of myeloid HDAC3 is neurovascular protective and leads to the resolution of retinal IR injury. The underlying mechanism may involve enhanced efferocytosis at the site of injury that promotes the removal of dead cells, which otherwise can contribute to inflammation. Interestingly, the pro-efferocytic condition caused by HDAC3 deletion appeared to be partially dependent on A1, implying that HDAC3 could also regulate efferocytosis by other unique pathways.

## Materials and methods

### Generation of myeloid-specific KO mice

#### Myeloid-specific HDAC3 KO mice

C57BL/6J floxed mice with LoxP sites on either side of exon 7 of HDAC3 (HDAC3^f/f^, originally developed by Dr. Scott W. Hiebert) were obtained from Dr. McGee-Lawrence and bred in our colony [34]. These mice were crossed with LysM Cre mice (Jackson Laboratory Stock No. 004781) to generate MΦ and microglia cell-specific HDAC3 KO (LysM Cre; HDAC3^f/f^ or M-HDAC3^−/−^) mice. Mice genotyping and characterization are shown in Supplementary Fig. S1.

#### Myeloid-specific A1 KO mice

C57BL/6J floxed mice that have LoxP sites for A1 (A1^f/f^) were crossed with LysM Cre mice (Stock No. 004781) to generate myeloid-specific A1 KO (M-A1^−/−^) mice, which were used for studies with isolated MΦ.

### Mouse retinal ischemia-reperfusion (IR) injury model and RGFP966 administration

All experiments were approved by the UAMS Institutional Animal Care and Use Committee (IACUC). M-HDAC3^−/−^, HDAC3^f/f,^ and wild-type (WT) C57BL/6J mice (10–12 weeks old) were anesthetized using a ketamine/xylazine mixture and then subjected to retinal ischemia followed by reperfusion to achieve IR injury as described by us and others [10, 35–37]. A needle connected to a raised saline bag was inserted into the anterior chamber of the right eye to raise the intraocular pressure to 110 mmHg (calculated based on the height of the saline bag). Ischemia was induced for 60 min followed by needle removal to allow reperfusion. The left eye served as a sham control since we did not detect differences between contralateral eyes and those from uninjured mice (Supplementary Fig. S2) [10, 37]. Animals with a drop in pressure due to saline leakage out of the eye during the procedure were excluded. Mice were deeply anesthetized and sacrificed by transcardial perfusion or cervical dislocation at the various time points shown in Table 1 and Supplementary Fig. S3 based on our previous studies and existing literature.

**Table 1:** Time points of the main outcome measures in the in vivo IR studies

Outcome measure	Time post-IR	References
Vascular permeability	2 days	[35, 38, 39]
Retina thickness measurements (OCT)	7 days	[40, 41]
Neurodegeneration	7 days	[35, 41, 42]
Retina function (ERG)	14 days	[36, 43]
Vascular degeneration (Trypsin digests)	14 days	[35, 42]

For studies on WT mice, the specific HDAC3 inhibitor RGFP966 (Sigma, St. Louis, MO) was dissolved in DMSO and then diluted in 70% polyethylene glycol (PEG) 200, 30% acetate buffer for intraperitoneal (i.p.) injection as previously described for a final DMSO concentration of ∼ 10% [44]. RGFP966 was administered (10 mg/kg i.p.) at 1 h after IR and every 48 h thereafter based on previous literature [45].

### Optical coherence tomography (OCT)

The eyes of mice were dilated with 1% tropicamide (Akron Pharmaceuticals, IL) and retinas were scanned with an OCT Ophthalmic Imaging System (Bioptigen Inc., Durham, NC) under ketamine/xylazine anesthesia. Mice were placed on a mouse holder to fix the animal’s posture for scanning. The retina scans were acquired in a rectangular volume mode (3 frames/scan, 1000 A scan/B scan × 100 B scan, 1.4 mm × 1.4 mm). Automated analysis of scans to obtain retinal thicknesses was conducted using InVivoVue software (Bioptigen Inc., Durham, NC).

### Electroretinography

A full scotopic electroretinogram (ERG) was recorded on day 14 post-IR as previously described with some modification [46]. Briefly, after 16 h of dark adaptation, mice were anesthetized using a ketamine/xylazine mixture, and the pupils were dilated with 1% tropicamide (Akron Pharmaceuticals, IL) eye drops. Mice were then placed on the Celeris electroretinogram system (Diagnosys LLC, Cambridge, UK) with temperature control (37 °C), and dark-adapted ERGs were recorded. Amplitudes and implicit times of ERG waveforms were measured at a series of flash intensities (0.01, 0.1, 1 cd.s/m^2^) based on a standard machine protocol and literature [46].

### Retinal vascular permeability

#### Albumin extravasation

Vascular permeability was examined by measuring the extravasation of albumin to the retina on day 2 after IR injury following transcardial perfusion to remove intravascular albumin as we and others previously described [35, 47]. Briefly, a 20-gauge perfusion cannula was introduced into the left cardiac ventricle of the anesthetized mice. Mice were perfused with phosphate-buffered saline (PBS) for 5 min to rinse out blood cells and proteins. Drainage of the blood in the PBS was conducted by opening the right atrium. Retinas were then lysed, and Western blotting was performed to quantify retinal vascular leakage by measuring extravascular albumin in the whole retina.

#### Retinal fluorescein angiography

Retinal vascular permeability in vivo was assessed using fluorescein angiography as previously described [48, 49]. Briefly, two days post-IR injury, mice were anesthetized using a ketamine/xylazine mixture, and pupils were dilated with 1% tropicamide (Akorn Pharmaceuticals, IL) eye drops. The anesthetized mice were mounted on a maneuverable imaging platform, and their corneas were lubricated with a thin layer of GenTeal moisturizing eye gel (Alcon, Geneva, Switzerland) to keep surface moisture during the procedure. Each mouse received a subcutaneous injection of 50 µL of AK-FLUOR^®^ fluorescein sodium (10%; Long Grove Pharmaceuticals, Rosemont, IL). Rapid image acquisition ensued over a period of approximately 5 min. Fluorescein leakage was demonstrated by indistinct vascular borders progressing to diffusely hazy fluorescence.

#### Evans blue leakage

In vivo retinal vascular permeability was assessed by imaging of intravascular Evans Blue dye extravasation at day two post-IR injury as previously described with some modification [50]. Briefly, anesthetized mice received transcardial injections of Evans Blue (200 µL, 2% in normal saline), euthanized after 5 min while under anesthesia, and eyes were fixed with 4% paraformaldehyde (PFA) for 24 h. Retinal flat mounts were mounted and cover-slipped with Vectashield antifade mounting medium (Vector Laboratories, Newark, CA), and images were captured using a fluorescence microscope. Images of the entire retina flat mount were processed using ImageJ. The red color (Evans Blue) was thresholded in each image, the background was subtracted, and the fluorescence area was binarized. Finally, the leakage area, represented as a binary area in the entire retina, was measured using ImageJ.

### Retinal vasculature trypsin digestion and counting of acellular capillaries

Eyeballs were isolated on day 14 after IR injury and fixed overnight in 4% PFA. The retinal vasculature was isolated by trypsin digestion as we and others have reported [6, 35, 51]. The vasculature was air-dried on silane-coated slides and stained with periodic acid-Schiff and hematoxylin. Acellular capillaries were counted in random fields of the mid-retina under the microscope. The number of acellular capillaries was divided by the field area to get the number of acellular capillaries per 1 mm of the retina [2].

### Fluorescent immunolabeling

Eyeballs were fixed in 4% PFA and then dissected into retinal flat mounts. The flat mounts were permeabilized in 0.1% triton X-100 and then blocked in 10% donkey serum and 3% bovine serum albumin (BSA) for 30 min. Subsequently, the flat mounts were incubated in primary antibodies including Iba1 (FUJIFILM Wako, Cat. #019-19741) and NeuN (MilliporeSigma, Cat. #BN78MI) overnight at 4 °C followed by washing in PBS and incubation of secondary antibodies at room temperature for 4 h as described earlier [35].

### Confocal microscopy and image analysis

Confocal imaging was performed using an LSM 880 Airyscan Zeiss Laser inverted microscope equipped with 405 nm, 488 nm, 561 nm, and 640 nm laser lines. Identical laser intensity settings were applied to all samples and Z-stacks images (resolution: 1024 × 1024 Pixels) were taken. Neurodegeneration and myeloid cell proliferation were determined as the NeuN^+^ and Iba1^+^ area, respectively [52]. After the acquisition, a maximum intensity projection of the Z-stack was applied using ZEN Blue software (Zeiss) or ImageJ. Quantitative analysis was performed using ZEN software or ImageJ on single-slice confocal images. Morphological analysis of myeloid cells utilized Sholl analysis (Fiji Sholl analysis plugin), as previously described [53]. Random Iba1^+^ cells in the ganglion cell layer (GCL) were chosen from three different fields of view and subjected to Sholl analysis. Criteria for cell selection included: (i) non-overlapping with other labeled cells, ensuring clear distinction of cell body and processes from neighboring cells, and (ii) all cellular processes within the field of view. The analysis determined the complexity of myeloid (Iba1+) cell processes by analyzing branch intersections at increasing radial distances from the cell body.

### Microscopy-based in vivo efferocytosis assay on retinal flat mounts

For microscopy-based quantification of in vivo efferocytosis, double immunolabeling of the microglia/MΦ marker, Iba1, and TUNEL staining of retinal flat mounts was conducted on day 2 after IR injury using the Click-iT™ Plus TUNEL Assay (Invitrogen™) following the manufacturer’s instructions. The number of Iba1^+^ positive cells associated with TUNEL^+^ apoptotic bodies was counted in multiple fields of view in Z-stack images taken at the ganglion cell layer using an LSM 880 Zeiss confocal microscope (Supplementary Fig. S4A). The efferocytosis index (% of dead/dying cells engulfed by myeloid cells) was calculated using the following equation: ([number of Iba1^+^TUNEL^+^ cells ÷ total number of TUNEL^+^ cells] × 100).

### Flow cytometry analysis of retinal immune cell populations

Following animal euthanasia by transcardial perfusion, eyes were removed by orbital dissection and retinas were isolated. Retinal tissues (2 to 4 retinas pooled per preparation) were processed for flow cytometry and analysis as described by us [54]. Briefly, retinas were digested in a solution containing 5% filtered fetal bovine serum (FBS, Gibco; Thermo Fisher, NY) 10 mM HEPES, 0.5 mg/mL of liberase (Sigma Aldrich, St. Louis, MO ), and 0.1 mg/mL of DNase (Sigma Aldrich, St. Louis, MO). Cells were strained through a 40 μm cell strainer and then incubated with a viability dye (Fixable Viability Dye eFluor™ 450, eBioscience™) for 30 min. After rinsing the viability dye, cell suspensions were blocked with 1 µg/mL of Fc block anti-mouse CD16/32 and 20% normal rat serum for 10 min at room temperature. Subsequently, retinas were incubated with labeled antibodies that included PerCP-Cy5.5-conjugated rat anti-mouse CD11b monoclonal antibody (1:100, Clone M1/70, BD Bioscience, Cat. #BDB550993), APC-Cy7 rat anti-mouse CD45 monoclonal antibody (1:100, Clone 30-F11, BD Bioscience, Cat. #BDB557659), PE-conjugated rat anti-mouse Ly6C monoclonal antibody (1:100, Clone HK1.4, eBioscience, Cat. #50-245-507), and FITC-conjugated rat anti-mouse Ly6G (Gr-1) monoclonal antibody (1:100, Clone RB6-8C5, eBioscience, Cat. #50-991-9). After incubation with antibodies, retinas were rinsed 3 times with cold PBS, fixed with 0.4% PFA, and analyzed using a BD LSRFortessa flow cytometer (BD Biosciences) and FlowJo software (Tree Star Inc., San Carlos, CA). Retinal cell populations initially were gated using the common leukocyte marker CD45 and the myeloid lineage marker CD11b. Based on the expression of CD45, myeloid cells were gated as CD11b^+^/CD45^low^ microglia and CD11b^+^/CD45^hi^ myeloid leukocytes. Myeloid leukocytes were gated further based on Ly6C expression and the granulocyte/neutrophil marker Ly6G. Myeloid leukocytes were subdivided into classical monocytes (CD11b^+^/CD45^hi^/Ly6C^hi^/Ly6G^neg^), intermediary monocytes (CD11b^+^/CD45^hi^/Ly6C^low^/Ly6G^neg^), and non-classical monocytes (CD11b^+^/CD45^hi^/Ly6C^neg^/Ly6G^neg^).

### Flow cytometry-based in vivo efferocytosis assay

In subsequent experiments, mice were treated with PSVue 550 eyedrops (Molecular Targeting Technologies, Cat. #P-1005) one day before sacrifice to assess efferocytosis by flow cytometry (Supplementary Fig. S4B). PSVue dye allows in vivo fluorescent labeling of dead cells since it binds to externalized phosphatidylserine (PtdSer, an “eat-me” signal) on apoptotic cells in live mice [55]. Retinas were then incubated with labeled antibodies that included PerCP-Cy5.5-conjugated rat anti-mouse CD11b monoclonal antibody (1:100, Clone M1/70, BD Bioscience, Cat. #BDB550993), APC-Cy7 rat anti-mouse CD45 monoclonal antibody (1:100, Clone 30-F11, BD Bioscience, Cat. #BDB557659) and eFluor 660 conjugated CD68 monoclonal antibody (eBioscience, Cat. #50-0681-82) with the latter used as a marker of phagocytic cells. Phagocytic microglia and myeloid leukocytes were gated from CD11b^+^/CD45^low^ and CD11b^+^/CD45^hi^ populations, respectively, as CD68^+^/PSVue^+^ cells engaged in efferocytosis of apoptotic cells.

### Cell isolation, cell lines, and culture

#### Bone marrow-derived macrophages (BMDMs)

Bone marrow cells were isolated and differentiated into MΦ based on our published protocol [6]. In brief, both femurs and tibias were harvested and flushed with 20 − 25 ml sterile PBS using a 27-gauge needle. Flushed cells in PBS were spun down and resuspended in differentiation medium (Dulbecco’s modified Eagle’s medium, DMEM, high glucose, Gibco; Thermo Fisher, NY)) containing 20% FBS (Gibco; Thermo Fisher, NY), 20% L929 conditioned media, and 1% penicillin-streptomycin (Pen-Strep). Cells were subsequently plated on uncoated 100-mm dishes. For studies of in vitro efferocytosis, BMDMs were plated on 35-mm glass bottom dishes (MatTek Corporation, Ashland, MA) with a 14-mm glass diameter. Media were replaced with fresh differentiation media on day 4 after plating.

#### K562 lymphoblast cells

Suspended K562 cells were cultured in RPMI 1640 medium (Gibco; Thermo Fisher, NY) containing 2 mM L-glutamine supplemented with 10% FBS (Gibco; Thermo Fisher, NY) and 100 IU/ml Pen-Strep (Gibco; Thermo Fisher, NY), and maintained in a 5% CO_2_ incubator at 37 °C [56].

#### R28 retinal neuronal-like cells

The R28 cell line (Kerafast, Boston, MA) was maintained and differentiated as described previously [52]. Cells were cultured in DMEM supplemented with 10% FBS (Gibco; Thermo Fisher, NY), 100 U/ml Pen-Strep, and maintained under standard culture conditions (37 °C, 5% CO_2_). The medium was changed completely every other day and cultures were passaged at 90% confluence. To induce differentiation, cells were passaged onto laminin-coated plates and supplemented with 250 µM 8-(4-Chlorophenylthio)adenosine 3′,5′-cyclic monophosphate sodium salt (pCPT-cAMP) for overnight incubation.

### Induction of apoptosis in K562 and R28 cells in vitro

K562 cells or R28 cells (apoptotic and non-apoptotic) were labeled with the Vybrant^®^ CFDA SE Cell Tracer Kit (Invitrogen, Carlsbad, CA) for 30 min at 37 °C, washed twice with complete media, and resuspended at 5 × 10^5^ cells/ml. BMDM were labeled with CM-DiI Dye^®^ (Invitrogen, Carlsbad, CA) in pre-warmed PBS and incubated for 15 min at 37 °C followed by another incubation for 30 min at 37 °C with DMEM (supplemented with 100 IU/ml Pen-Strep) before rinsing three times with PBS.

Apoptosis of K562 or R28 cells was induced by subjecting the cells to UV-B irradiation for 15 min using a UV crosslinker (Spectrolinker™ XL-1500, Spectronics Corporation, Melville, NY). Afterward, the cells were resuspended in DMEM containing 10% FBS (Gibco; Thermo Fisher, NY) and incubated at 37 °C for up to two hours and monitored for apoptosis induction (visible cell blebbing under the microscope). Apoptosis was also confirmed by Annexin V labeling (Supplementary Fig. S4C) [56]. UV-B was chosen as the apoptosis induction method due to its rapid action and widespread use [56, 57].

### Evaluation of macrophage efferocytosis of apoptotic cells in vitro

Three sets of experiments using the K562 and R28 cells prepared as described above were conducted to evaluate in vitro efferocytosis:

#### Experiment 1

CM-DiI labeled BMDMs derived from HDAC3^f/f^ and M-HDAC3^−/−^ mice were plated on 35-mm dishes and treated with or without the A1 inhibitor, 2(S)-amino-6-boronohexanoic acid (ABH) (100 µM). Then cells were incubated with CFDA-labeled K562 cells (apoptotic or non-apoptotic) at a 1:1 ratio for 45 min at 37 °C followed by PBS washing to remove non-engulfed K562 cells. Subsequently, the dishes either were imaged under a confocal microscope to detect the efferocytic MΦ or were trypsinized and immediately analyzed by the BD Accuri™ C6 Plus Flow Cytometer (BD Bioscience, San Jose, CA). Cells that were double positive for CM-DiI/CFDA were regarded to represent phagocytic BMDMs that engulfed apoptotic cells. Efferocytosis was calculated as a fold change by dividing CM-DiI^+^ CFDA^+^ (phagocytic BMDMs) by the total number of CM-DiI^+^ BMDMs.

#### Experiment 2

CM-DiI labeled BMDMs derived from A1^f/f^ and M-A1^−/−^ mice and plated on 35-mm dishes were incubated with CFDA-labeled R28 cells (apoptotic or non-apoptotic) for 45 min at 37 °C followed by PBS washing to remove non-engulfed cells. The dishes were then processed for imaging under the microscope to detect the efferocytic MΦ.

#### Experiment 3

CM-DiI labeled BMDMs derived from A1^f/f^ and M-A1^−/−^ mice that were plated on 100-mm dishes were incubated with CFDA-labeled K562 cells (apoptotic and non-apoptotic) for 45 min at 37 °C. After washing the non-engulfed cells, the adherent BMDMs either were imaged by a confocal microscope or trypsinized and immediately analyzed by flow cytometry.

### Real time-PCR to detect A1 expression in BMDMs

For RNA isolation, 4 × 10^5^ BMDMs derived from HDAC3^f/f^ and M-HDAC3^−/−^ mice were seeded onto 6-well plates and incubated with 7.5 × 10^5^ K562 cells (apoptotic or non-apoptotic) for 45 min at 37 °C. Following treatment, K562 cells were removed by several PBS washes. BMDMs were then incubated for another 5 hours, and total RNA was isolated using TRIzol reagent (Invitrogen, CA) and converted to cDNA using M-MLV reverse transcriptase (Invitrogen, CA). The reverse-transcriptase qPCR assay was performed using a Verso cDNA Synthesis Kit (Fisher Scientific, NJ). Gene expression was performed using the PowerTrack™ SYBR Green Master Mix (Applied Biosystems™) and a CFX96 Touch Real-Time PCR Detection System (BioRad). Data were analyzed using the comparative Ct method with GAPDH as the reference gene. For A1, the following primer sequences were used: Forward, CAGAAGAATGGAAGAGTCAG; Reverse, CAGATATGCAGGGAGTCACC.

### Preparation of BMDMs subjected to efferocytosis for Western blotting

BMDMs derived from HDAC3^f/f^ and M-HDAC3^−/−^ mice were seeded into 6-well plates and incubated with 7.5 × 10^5^ K562 cells (apoptotic or non-apoptotic) for 45 min at 37 °C. BMDMs were then washed several times with PBS to rinse the non-engulfed K562 cells and further incubated for 18 h before protein extraction in RIPA buffer prior to Western blotting.

### Western blotting of frozen retinas and cells

Retinas or cells were collected, snap-frozen, and then stored in a -80^o^C freezer for further processing. Tissues or cells were homogenized in RIPA buffer (Thermofisher) and Western blotting was conducted as previously described [6]. The primary antibodies were as follows: HDAC3 (BD Bioscience, San Jose, CA, Cat. #611,124), albumin (Proteintech, Cat. #16475-1-AP), A1 (GeneTex, Cat. #GTX109242), and β-actin (Sigma, Cat. #A5441). Secondary antibodies (Invitrogen) were prepared in 5% milk in a 1:2000 dilution.

### Human eye sections and transcriptome

Postmortem human eye paraffin-embedded sections were obtained from the National Disease Research Interchange (NDRI, Philadelphia, PA). Sections from patients diagnosed with DR and controls were deparaffinized in xylene (Fisher Scientific, NJ), and rehydrated in graded ethanol baths (100%, 90%, 70%, 50%) followed by a final incubation in distilled water. Antigen retrieval was achieved by microwaving the sections in Tris-EDTA buffer (10 mM Tris Base, 1 mM EDTA, 0.05% Tween 20, pH 8.0). Sections were incubated in 0.2% Triton X-100 in PBS for 15 min followed by blocking with 3% normal donkey serum (MilliporeSigma, Cat. # 5,058,837) and 3% BSA for 1 h. Sections were then incubated overnight at 4 °C with diluted primary antibodies in a blocking buffer that included Iba1 (FUJIFILM Wako, Cat. # 011-27991), and HDAC3 (Invitrogen, Cat. #PA5-29026). After primary antibody incubation, sections were incubated with the appropriate secondary antibody, then washed with cold PBS and cover-slipped with Vectashield antifade mounting medium containing 4′,6-diamidino-2-phenylindole (DAPI; Vector Laboratories, Newark, CA) to mark nuclei.

Information on HDAC3 expression in the human eye was obtained by searching the Human Eye Transcriptome Atlas, a publicly available database https://www.eye-transcriptome.com/search_latest.php.

### Statistical analysis

Statistics were performed and graphs prepared using GraphPad Prism 10 software and data were presented as mean ± standard deviation (SD). A *p*-value < 0.05 was considered statistically significant. All statistical analyses were performed using the Student’s *t-*test (for two group comparisons) or analysis of variance (ANOVA) with Tukey’s post hoc test (for comparison of multiple groups).

## Results

### Deletion of myeloid HDAC3 protects retinal neurons from IR injury and preserves retinal thickness

**Fig. 1:**
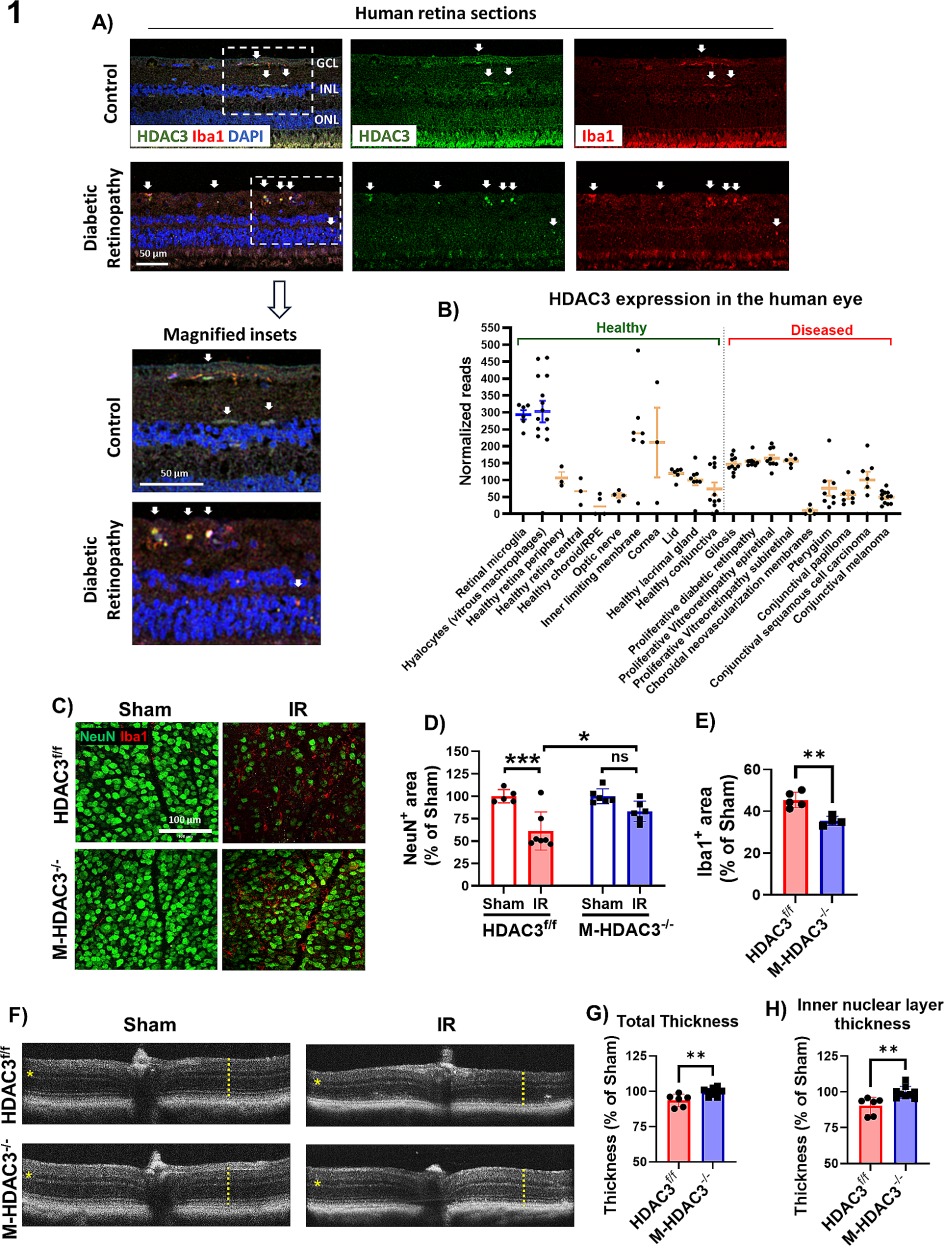
Myeloid HDAC3 deletion is neuroprotective and mitigates retinal thinning after IR injury. (**A**) Immunolabeling of postmortem human retina sections from control and DR patients show colocalization of HDAC3 with Iba1^+^ myeloid cells. GCL: ganglion cell layer, INL: inner nuclear layer, ONL: outer nuclear layer, *n* = 3. (**B**) Graph extracted from a searchable transcriptome database for human eye tissue (https://www.eye-transcriptome.com/) shows high HDAC3 expression in retinal microglia and vitreous MΦ. **C-E**) Immunolabeling at 7 days after injury and quantification show reduced neurodegeneration (marked by the neuronal marker, NeuN) and microglia/MΦ numbers (marked by Iba1) in the M-HDAC3^−/−^ retinas as compared to HDAC3^f/f^, *n* = 5–9. **F-H**) Optical coherence tomography (OCT) performed on anesthetized mice 7 days after IR shows preserved total retinal thickness (indicated by a dashed line) and preserved thickness of the inner nuclear layer (indicated by a star) in M-HDAC3^−/−^ retinas, *n* = 6–9, *p < 0.05, **p < 0.01, ***p < 0.005, ns: not significant

We recently reported that HDAC3 is upregulated in murine retinal microglia/MΦ following IR injury [5]. Here, we confirm this preclinical finding in human retina sections from patients with DR, a disease characterized by varying degrees of retinal ischemia. HDAC3 showed expression in Iba1^+^ myeloid cells in retina sections from control subjects and patients with DR with the latter showing amoeboid myeloid cells with strong expression of HDAC3. (Fig. 1A). Searching a publicly available eye transcriptome database showed the highest HDAC3 expression in retinal microglia and vitreous MΦ (Fig. 1B).

To investigate the role of myeloid HDAC3 in retinal injury, we generated a myeloid-specific HDAC3 KO mouse line using a LysM Cre driver (characterized in Supplementary Fig. S1). This Cre driver deletes the gene of interest in MΦ and ∼ 30% of microglia [58]. We subjected myeloid HDAC3 KO mice (M-HDAC3^−/−^) and floxed controls (HDAC3^f/f^) to 60 min of ischemia followed by reperfusion (IR) injury. A series of assays to evaluate retinal injury outcomes were conducted at different time points based on our previous work (Supplementary Fig. S3 and Table 1 depict the experimental timeline) [6]. We initially examined neurodegeneration and myeloid cell proliferation at day 7 after IR by immunolabeling retina flat mounts with the neuronal marker NeuN and the microglia/MΦ marker Iba1 using our established protocol [6]. Retinas from IR-injured floxed control mice showed neuronal degeneration and myeloid cell activation as evidenced by decreased NeuN and increased Iba1 labeling, whereas retinas from mice lacking myeloid HDAC3 showed neuronal preservation after IR and decreased Iba1 labeling (Fig. 1C-E). The Sholl analysis on day 2 after IR revealed that myeloid cells from the IR-injured groups exhibited reduced branching complexity compared to sham controls. Interestingly, there were no significant differences in branching complexity within either the IR or sham groups (Supplementary Fig. S5). OCT at day 7 after IR showed preservation of retinal thickness in the M-HDAC3^−/−^ mice compared to the injured HDAC3^f/f^ controls (Fig. 1F-H). Collectively, these data suggest that myeloid HDAC3 contributes to retinal neuronal degeneration following IR injury and that HDAC3 deletion mitigates IR-induced retinal degeneration and thinning.

### Deletion of myeloid HDAC3 preserves retinal function after IR injury

**Fig. 2:**
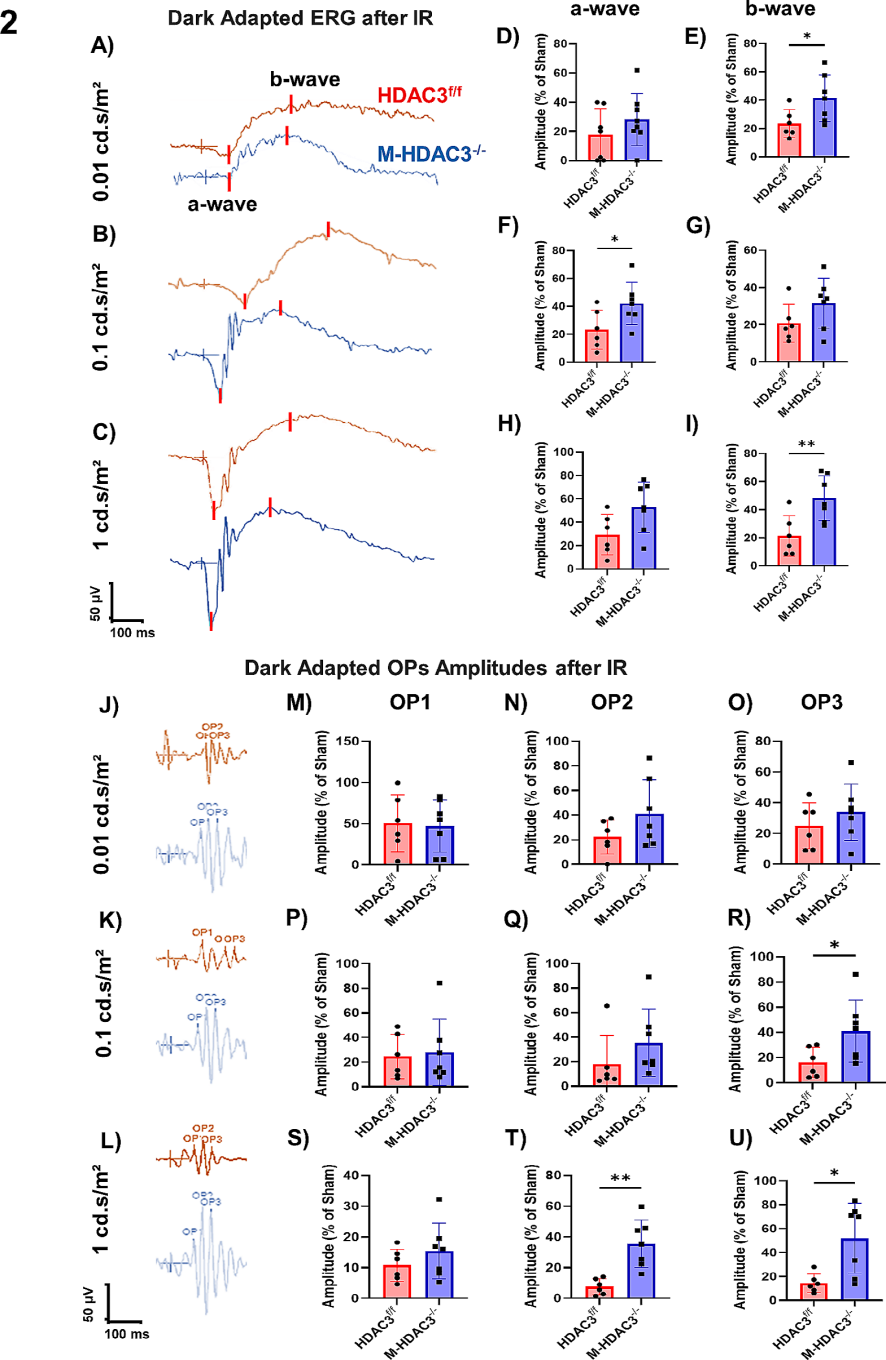
Myeloid HDAC3 deletion improves the retinal ERG after IR. **A-C**) Representative a- and b-waveforms elicited by 3 flash intensities in retinas of HDAC3^f/f^ and M-HDAC3^−/−^ mice on day 14 after IR injury. **D-I**) Quantification and comparison reveal improved waveform amplitudes in M-HDAC3^−/−^ retinas with statistical significance achieved at flash intensities of 0.1 cd.s/m2 (a-wave) and 0.01 cd.s/m^2^ and 1.0 cd.s/m² (b-wave). **J-L**) Representative waveforms of oscillatory potentials (OPs) in retinas of HDAC3^f/f^ and M-HDAC3^−/−^ mice elicited by 3 flash intensities at day 14 after IR injury. **M-U**) Quantification reveals a pattern of improved OP with statistical significance achieved at 1 cd.s/m² (OP2) and at 0.1 cd.s/m^2^ and 1.0 cd.s/m² (OP3), *n* = 6–8, *p < 0.05, **p < 0.01

To determine whether the retinal neuroprotection and structural preservation conferred by myeloid HDAC3 deletion translates to improved retinal function, we conducted electroretinographic (ERG) measurements on mice subjected to IR and then allowed to recover for 14 days. Three progressive flash intensities were used (Fig. 2A-C). Measurements included analysis of a-wave amplitude (a measure of photoreceptor function) and b-wave amplitude (a measure of ON bipolar cell function) and oscillatory potential amplitudes (OPs, including the three major wavelets, OP1, OP2, and OP3, and provide a measure of inner retina 3rd order neurons function). Uninjured M-HDAC3^−/−^ and HDAC3^f/f^ mice showed no difference in ERG response (Supplementary Fig. S6). IR injury of HDAC3^f/f^ retinas resulted in a marked reduction in a-wave amplitude at 14 days. This loss of function was partially ameliorated in injured retinas of M-HDAC^−/−^ mice in which a-wave amplitude was higher than in injured HDAC3^f/f^; statistical significance was reached at a flash intensity of 0.1 cd.s/m^2^ (Fig. 2D, F, H). Similarly, we observed a drastic reduction in b-wave amplitudes elicited by 3 flash intensities in injured HDAC3^f/f^ retinas subjected to ERG at 14 days. In comparison to HDAC3^f/f^ mice, the b-wave amplitude was higher in M-HDAC3^−/−^ retinas, reaching significance at the flash intensities of 0.01 cd.s/m^2^ and 1.0 cd.s/m² (Fig. 2E, G, I). M-HDAC3^−/−^ retinas showed better OPs only at higher flash intensities, 0.1 and 1 cd.s/m² (Fig. 2J-U). Of note, ERG response time was not different between the M-HDAC3^−/−^ and HDAC3^f/f^ IR groups (Supplementary Fig. S7). Collectively, the ERG data confirms the retinal neuronal function preservation after IR with myeloid HDAC3 deletion.

### Deletion of myeloid HDAC3 attenuates IR-induced retinal permeability and vascular degeneration

**Fig. 3:**
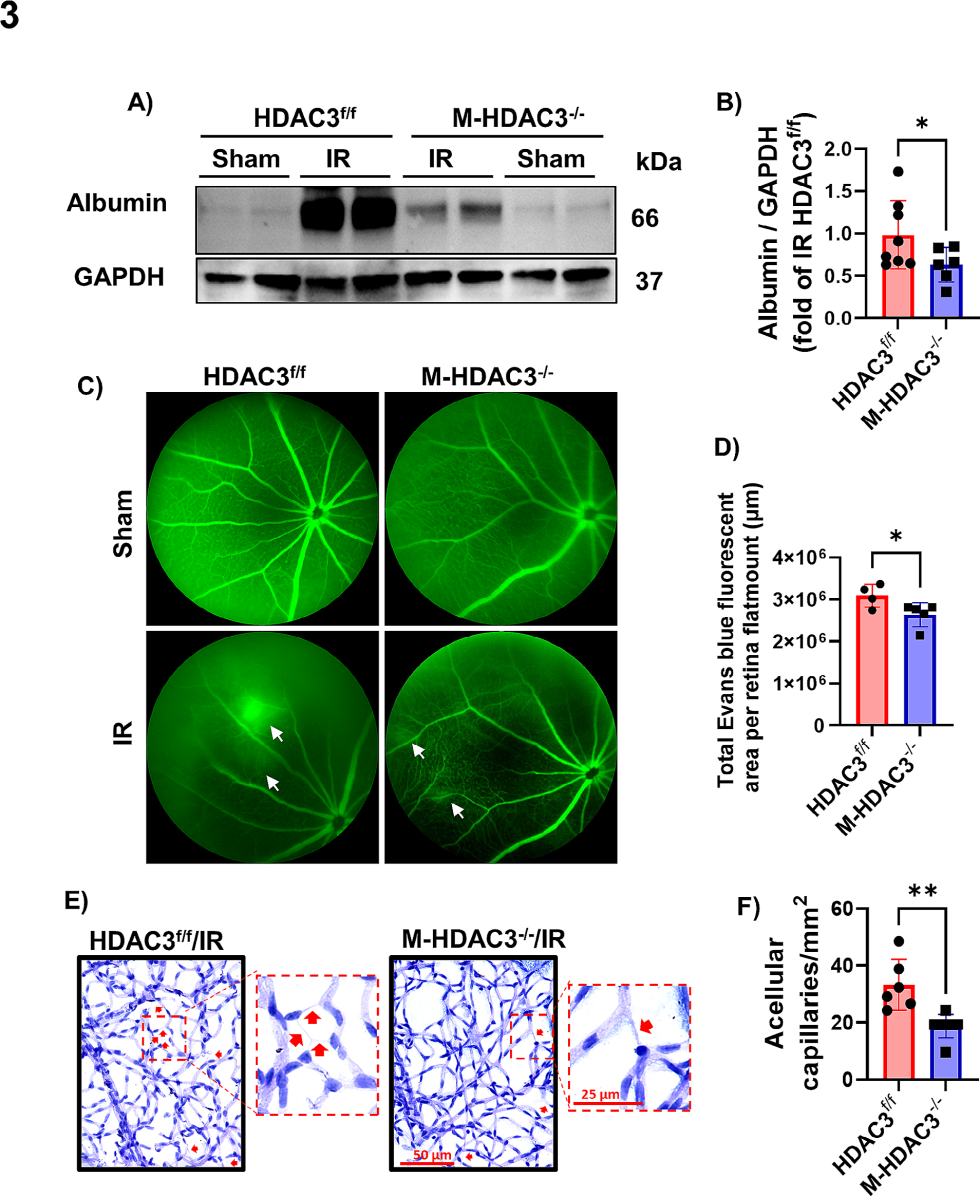
Myeloid HDAC3 deletion is vascular protective in retinal IR injury. **A, B**) Albumin extravasation at day 2 post-IR was measured in protein lysates of perfused retinas by western blot. Extravasated albumin was markedly reduced in M-HDAC3^−/−^ retinas compared to HDAC3^f/f^ controls and was nearly absent in the contralateral retinas. GAPDH was used as a loading control, *n* = 6–8. **C**) Representative retinal fluorescein angiography images at day two post-IR showing increased retinal vascular permeability. Leaked fluorescein demonstrated by diffusely hazy fluorescence (white arrows) was markedly reduced in M-HDAC3^−/−^ retinas compared to HDAC3^f/f^ controls and was absent in the sham retinas. **D**) Quantification of Evans blue leakage demonstrates a significant reduction in the M-HDAC3^−/−^ IR retinas, *n* = 4–5. Representative images are included in supplementary figure S8. **E, F**) Vascular digests at 14 days after IR injury showed increased numbers of acellular retinal capillaries (empty basement membrane sleeves - red arrows) in injured HDAC3^f/f^ retinas compared to injured M-HDAC3^−/−^ retinas, *n* = 6–8, *p < 0.05, **p < 0.01

Next, we examined the effect of myeloid HDAC3 deletion on IR-induced disruption of the blood-retinal barrier. An intact blood-retinal barrier relies on the integrity of tight junctions of the retinal vascular endothelium. Changes in barrier permeability in perfused mouse retinas on day 2 after IR were assessed by evaluating extravasated albumin on Western blot. Injured retinas from M-HDAC3^−/−^ mice were profoundly protected from blood-retinal barrier disruption compared to HDAC3^fl/fl^ retinas as evidenced by reduced albumin leakage (Fig. 3A, B). Reduced retinal permeability in M-HDAC3^−/−^ mice was confirmed using fluorescein angiography and Evans blue leakage (Fig. 3C, D, and Supplementary Fig. S8). Vascular integrity was further assessed by conducting trypsin digestion of retinas and counting acellular capillaries at day 14 after injury. Injured M-HDAC3^−/−^ retinas showed fewer acellular capillary formations compared to injured HDAC3^f/f^ retinas, confirming the vascular protective effect of myeloid deletion of HDAC3 (Fig. 3E, F).

### HDAC3 deletion ameliorates the myeloid cell response to retinal injury

**Fig. 4:**
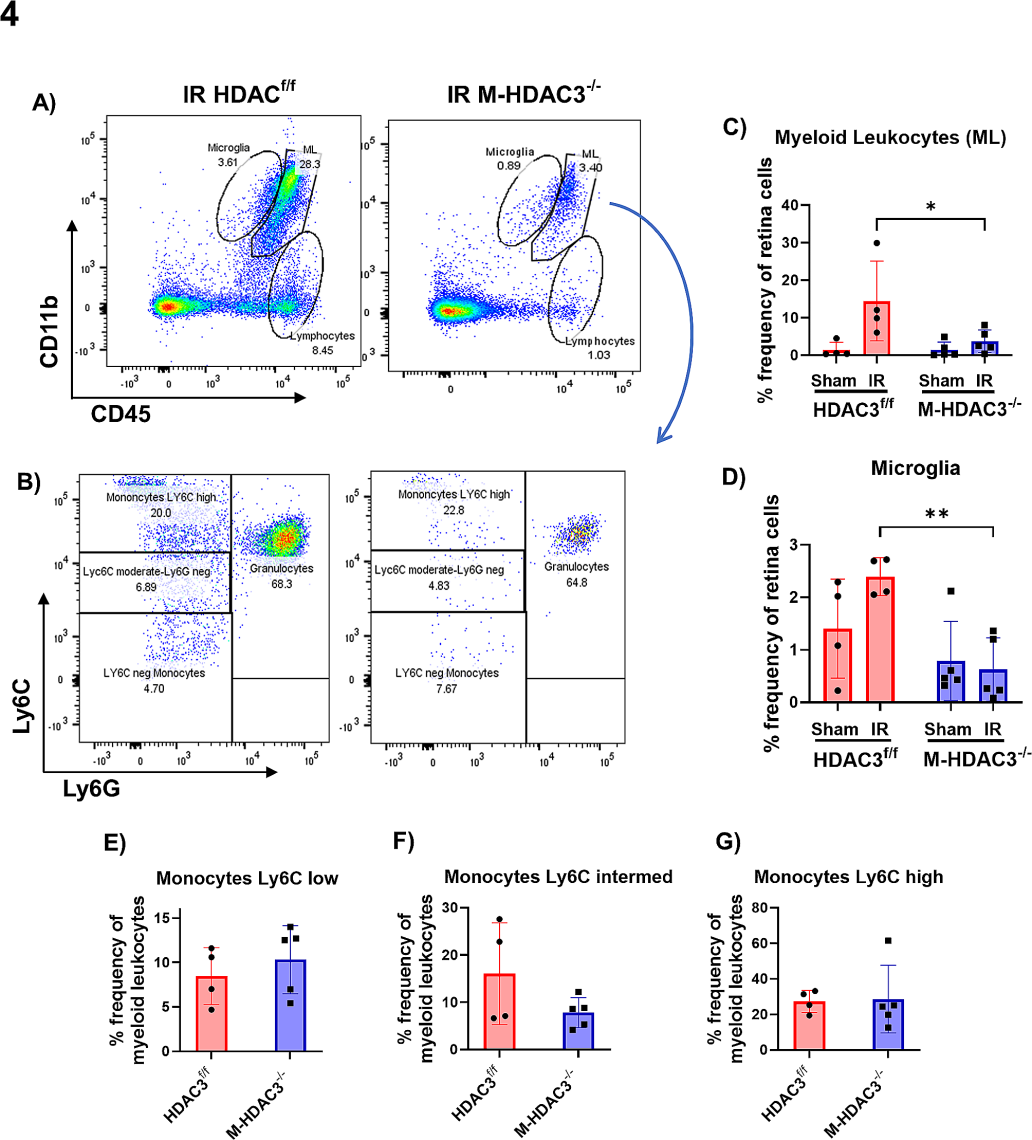
M-HDAC3 deletion mitigates the IR-induced microglia proliferation and myeloid leukocyte infiltration into the retina. (**A**) Representative scatter graphs from flow-cytometric analysis showing the initial gating strategy used to quantify the immune cell populations of microglia, myeloid leukocytes (ML), and lymphocytes in HDAC3^f/f^ and M-HDAC3^−/−^ retinas on day two after IR injury. (**B**) The ML were further gated based on Ly6C/Ly6G expression into Ly6C^hi^/Ly6G^neg^ monocytes, Ly6C^moderate^/Ly6G^neg^ monocytes, Ly6C^neg^/Ly6G^neg^ monocytes, and Ly6C^+^/Ly6G^+^ granulocytes. (**C**) The number of CD11b^+^/CD45^hi^ ML was reduced in injured M-HDAC3^−/−^ retinas compared to injured HDAC3^f/f^ controls indicating reduced myeloid cell infiltration/proliferation. (**D**) Similarly, the number of CD11b^+^/CD45^low^ microglia was reduced in injured M-HDAC3^−/−^ retinas. **E-G**) ML further gated into Ly6C^low^, Ly6C^intermediate^, and Ly6C^high^ monocytes showed no change in percentage frequency between the injured groups. For each data point, 3 retinas were pooled and *n* = 4–5 preparations of pooled retinas were analyzed for each group. *p < 0.05, **p < 0.01

Flow cytometric analysis of retina myeloid cells was conducted on day 2 after IR according to our established protocol [54]. Retina myeloid cells were gated using CD45 and CD11b markers and myeloid leukocytes were identified as CD11b^high^, CD45^high^, whereas microglia were identified as CD11b^high^, CD45^intermediate^ [54]. The IR injury led to a robust myeloid cell response in retinas (Fig. 4A, B) in agreement with previous work [10, 54]. Quantification of myeloid leukocyte and microglia showed a ∼ 10-fold increase in myeloid leukocyte number (Fig. 4C), whereas there was a trend toward increased microglia numbers (Fig. 4D). Interestingly, the strong increase in myeloid cells was dampened in the M-HDAC3^−/−^ retinas injured by IR. Notably, the frequency of Ly6C^low^ reparative monocytes, Ly6C^intermediate^ and Ly6C^high^ inflammatory monocytes was not significantly different between injured M-HDAC3^−/−^ and HDAC3^f/f^ retinas (Fig. 4E-G) despite the lower myeloid leukocytes count in the M-HDAC3^−/−^ retinas. These results indicate that HDAC3 deletion reduces myeloid cell proliferation/infiltration in the injured retina after IR.

### HDAC3 deletion increases myeloid cell efferocytosis activity

**Fig. 5:**
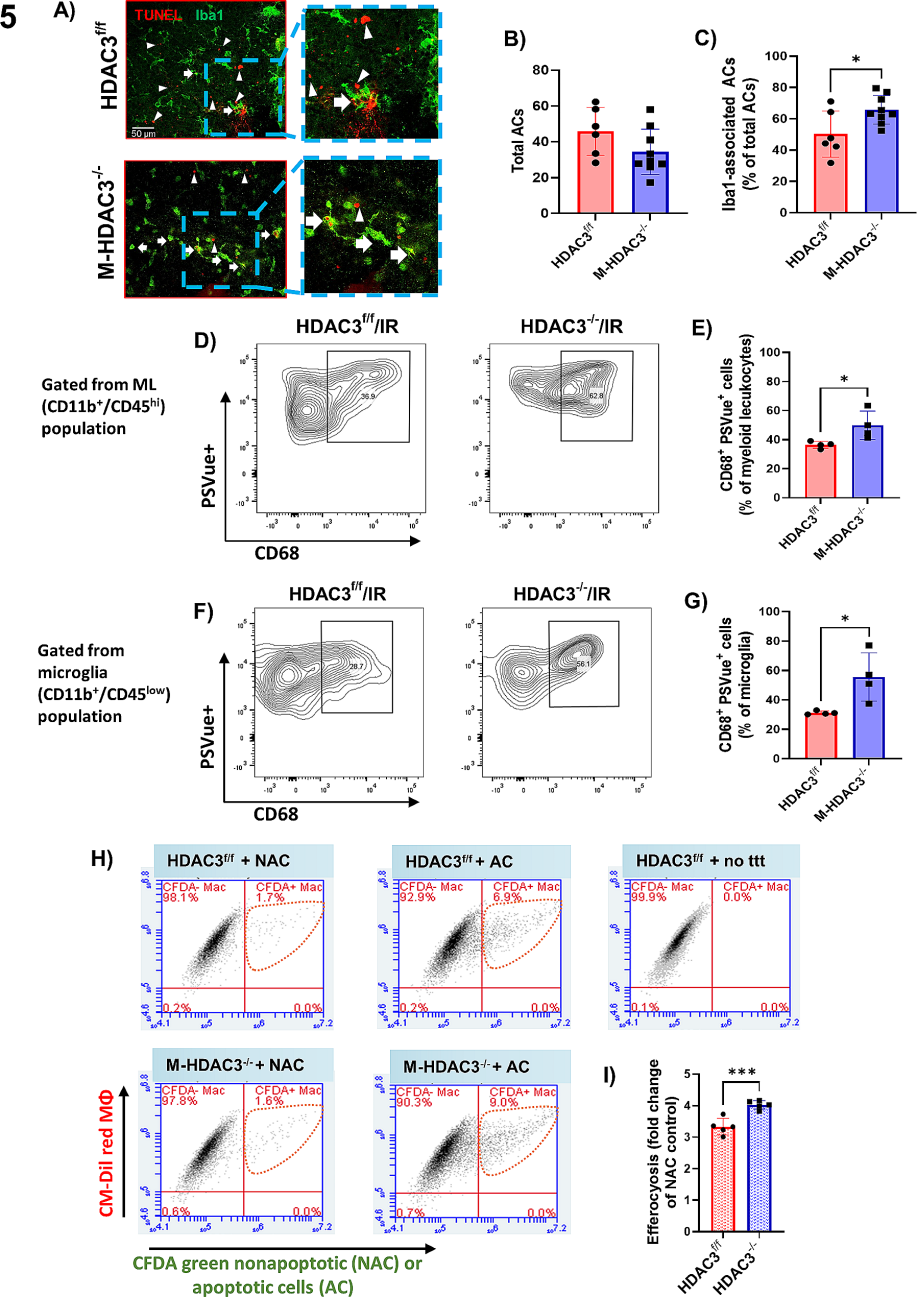
M-HDAC3 deletion improves macrophage clearance of apoptotic cells through efferocytosisin vivo and *in vitro.***A**) Representative confocal images of immunolabeled TUNEL^+^ apoptotic cells (red) and Iba1^+^ myeloid cells (green) in retinal flat mounts on day 2 after IR injury. Arrows and arrowheads depict Iba1-associated and free TUNEL^+^ cells, respectively. **B**) The total number of apoptotic cells (ACs, TUNEL^+^) was not significantly different between the injured groups. **C**) Quantification of the efferocytosis index (% Iba1^+^ ACs / total ACs) indicated enhanced phagocytosis in injured retinas of M-HDAC3^−/−^ compared to HDAC3^f/f^ mice, *n* = 6–8. **D-G**) Flow-cytometric analysis of myeloid leukocytes and microglia co-expressing the phagocytic marker CD68^+^ and the apoptotic cell signal PSVue reveals more CD68^+^/PSVue^+^ cells in retinas of M-HDAC3^−/−^ compared to HDAC3^f/f^ mice at day 2 after IR injury, *n* = 5 per group. **H**) Representative scatter plots from flow cytometry studies in which non-apoptotic (NAC) or apoptotic (AC) CFDA green-labeled K-562 cells were co-cultured with CM-DiI red-labeled MΦ. Control MΦ received no treatment (no ttt). **I**) Quantification by fluorescence-activated cell sorting (FACS) of MΦ engaged in efferocytosis of ACs (Dil^+^/CFDA^+^) as a percent of total MΦ followed by normalization to the NAC group. Efferocytosis was enhanced in HDAC3^−/−^ MΦ, *n* = 5 per group, *p < 0.05, ***p < 0.005. No-treatment group was used as a negative control for gating of CFDA- macrophages

Since HDAC3 deletion did not affect the myeloid cell inflammatory response as measured by Ly6C expression, we sought to investigate efferocytosis as a potential reparative mechanism employed by myeloid cells. We employed two distinct assays to evaluate whether deletion of HDAC3 increases MΦ-mediated reparative clearance of apoptotic cells by efferocytosis. Confocal microscopy was performed to detect co-localization of TUNEL-labeled apoptotic cells (ACs) with Iba1-labeled myeloid cells. We chose 2 days post-IR injury as the apoptosis peak at this time point based on our preliminary studies and published literature [59]. The total number of TUNEL^+^ cells in M-HDAC3^f/f^ and M-HDAC3^−/−^ retinas was not significantly different at 2 days post-IR injury. Interestingly, the M-HDAC3^−/−^ retinas showed increased Iba1^+^ myeloid cell association with TUNEL^+^ ACs as an indication of efferocytosis (Fig. 5A-C). Results from flow cytometry confirmed that M-HDAC3^−/−^ retinas exhibited higher levels of efferocytosis at 2 days after IR injury. In these studies, ACs were labeled in vivo using eye drops with PSVue 550 before retinas were removed and trypsin-digested for flow cytometry, which quantified myeloid leukocytes and microglia positive for the phagocytic marker CD68 that exhibited engulfed PSVue^+^ ACs (CD68^+^/PSVue^+^). M-HDAC3^−/−^ retinas displayed enhanced efferocytosis as measured by higher numbers of CD68^+^/PSVue^+^ myeloid leukocytes (Fig. 5D, E) and microglia (Fig. 5F, G). Collectively, these findings imply that HDAC3 inhibits efferocytosis since its deletion encourages this process.

We explored this possibility and underlying mechanisms using in vitro efferocytosis assays. We labeled HDAC3^−/−^ and HDAC3^f/f^ (control) MΦ with Dil-red fluorescent dye for identification and incubated them with CFDA green-labeled ACs, or non-apoptotic K562 lymphoblasts (NAC), or performed no cell treatment (no ttt). K562 cells are easily induced to be apoptotic and are routinely used in suspension cultures because they are readily washed from adherent MΦ, which enables accurate counting of the remaining engulfed K562 cells [7]. Flow cytometric analysis of phagocytic MΦ (Dil^+^/CFDA^+^) showed increased efferocytosis of ACs by HDAC3^−/−^ compared to HDAC3^f/f^ MΦ (Fig. 5H, I). Collectively, these results suggest the reparative effect of myeloid HDAC3 deletion involves enhanced efferocytic functions.

### The proefferocytic effect of HDAC3 deletion is partially dependent on arginase 1

**Fig. 6:**
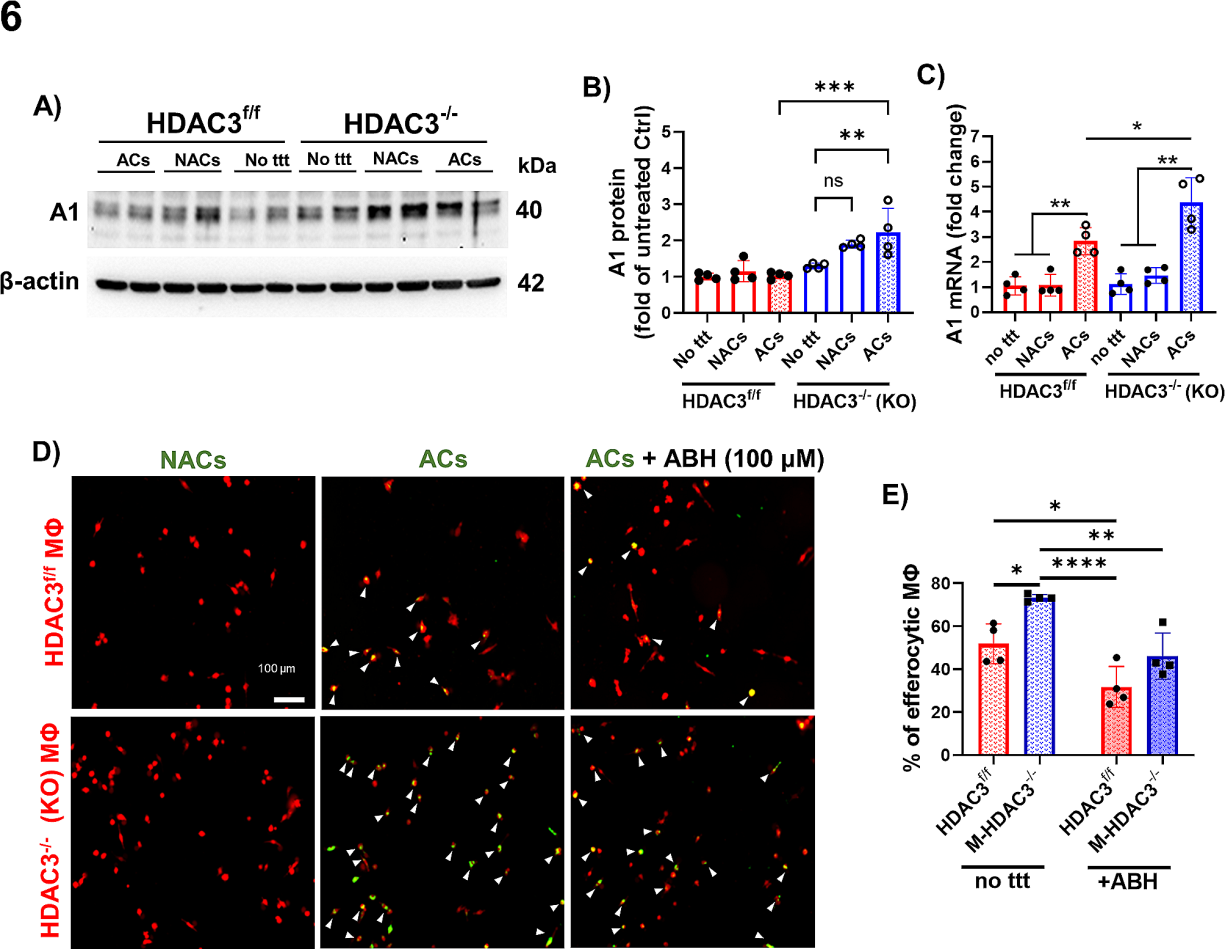
Arginase 1 is upregulated in M-HDAC3^−/−^macrophages and its inhibition impairs macrophage-mediated efferocytosis. **A, B)** Western blot shows upregulation of Arginase 1 (A1) in M-HDAC3^−/−^ but not HDAC3^f/f^ MΦ after co-incubation with K-562 ACs. Controls included either co-incubation of MΦ with K-562 NACs or no treatment (no ttt), *n* = 4. **C**) The corresponding A1 transcript was increased in M-HDAC3^−/−^ MΦ co-cultured with K-562 ACs as compared to control preparations. A1 transcript also increased in HDAC3^f/f^ MΦ co-cultured with ACs but significantly less than in AC/M-HDAC3^−/−^ MΦ co-cultures, *n* = 4. **D**)In vitro efferocytosis assay in which CFDA green-labeled K-562 NACs or ACs (shown in green) were added to DiI red-labeled MΦ (shown in red) after 45 min pre-incubation with the A1 inhibitor ABH (100 µM). Arrowheads indicate efferocytosis of ACs by HDAC3^f/f^ or M-HDAC3^−/−^ MΦ, *n* = 4. **E**) Quantification of MΦ engaged in efferocytosis of ACs (Dil^+^/CFDA^+^) as a percent of total MΦ reveals enhanced efferocytosis in M-HDAC3^−/−^ MΦ. Two-way ANOVA showed that the effect of HDAC3 deletion and ABH treatment were independently statistically significant but there was no interaction between the two, *n* = 4, *p < 0.05, **p < 0.01, ***p < 0.005, ****p < 0.001

**Fig. 7:**
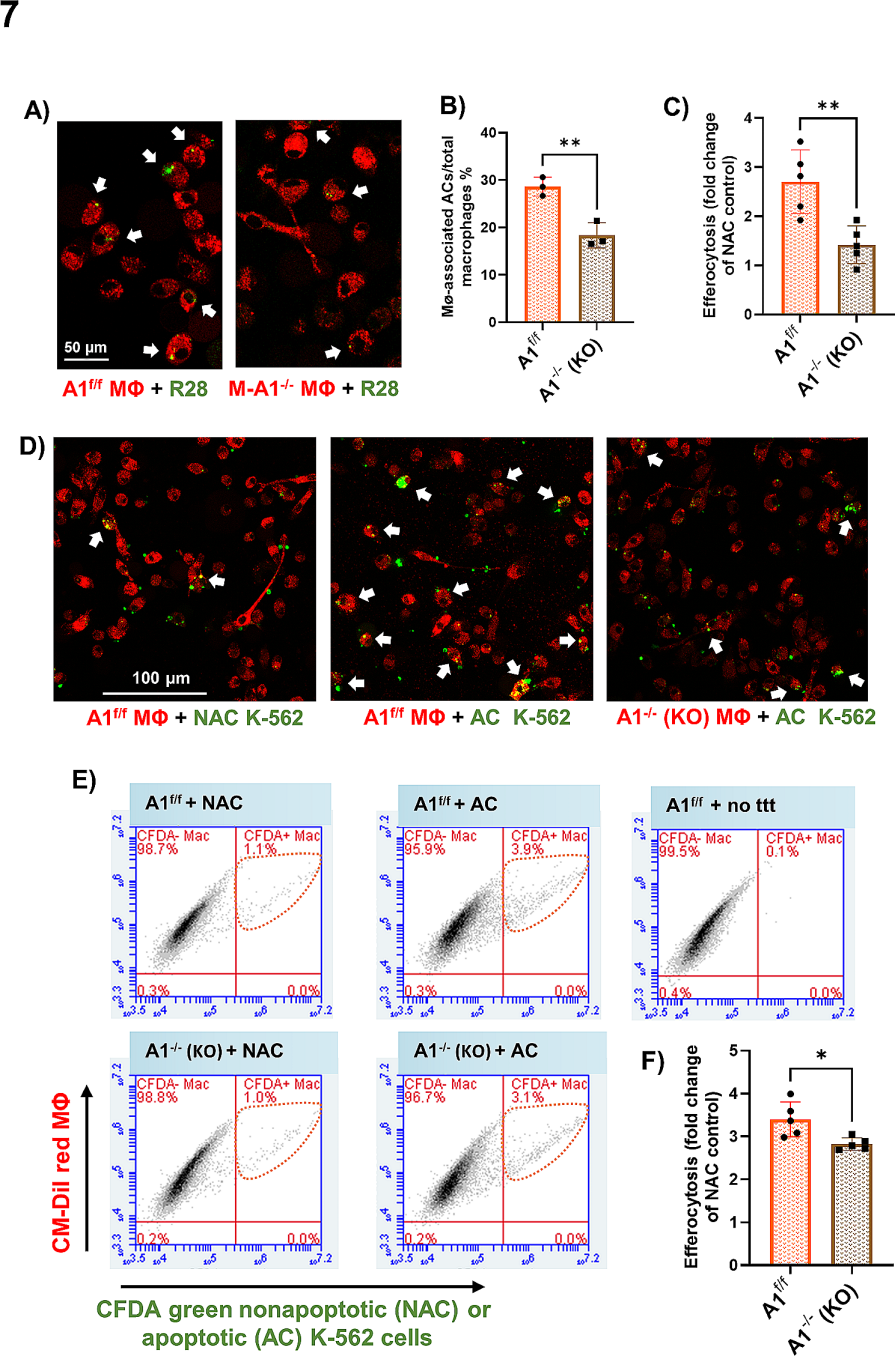
Arginase 1 deletion inhibits efferocytosis in macrophages (**A**) Representative images from an in vitro efferocytosis assay in which CFDA green-labeled R28 ACs were added to CM-DiI red-labeled MΦ isolated from M-A1^−/−^ or A1^f/f^ mice. (**B**) Efferocytosis was reduced in MΦ from M-A1^−/−^ mice, *n* = 3. **C, D**) A similar efferocytosis assay but using K-562 ACs also showed reduced efferocytosis by MΦ from M-A1^−/−^ mice compared to A1^f/f^ mice, *n* = 5 per group. **E, F**) Scatter plots and quantification from FACS of CM-DiI^+^/CFDA^+^ MΦ confirmed impaired efferocytosis of K-562 AC by M-A1^−/−^ compared to A1^f/f^ MΦ, *n* = 5 per group, *p < 0.05, **p < 0.01

We recently demonstrated that deletion of the enzyme A1 in MΦ increases the expression of HDAC3 [5]. Since a contribution of the A1 pathway to efferocytosis has been reported in rodent models of stroke and atherosclerosis [13, 32, 33], we explored the possibility that HDAC3 downregulates A1 in a negative feedback loop to limit efferocytosis. Indeed, the addition of apoptotic cells to HDAC3^−/−^ MΦ in our in vitro efferocytosis assay resulted in a marked increase in A1 protein (Fig. 6A, B) and transcript (Fig. 6C) that were significantly higher than in HDAC3^−/−^ MΦ treated with apoptotic cells. To determine whether HDAC3 deletion promotes A1-mediated efferocytosis, HDAC3^−/−^ MΦ and HDAC3^f/f^ MΦ were treated with the A1 inhibitor, ABH, and subjected to efferocytosis. As anticipated, HDAC3 deletion enhanced efferocytosis whereas A1 inhibition attenuated efferocytosis in HDAC3^f/f^ and HDAC3^−/−^ MΦ (Fig. 6D, E). However, two-way ANOVA statistical analysis showed no interactive effect between HDAC3 and A1, suggesting that the inhibitory effect of HDAC3 on efferocytosis is not solely dependent on A1 (Fig. 6D, E). To confirm that inhibition of A1 impairs efferocytosis, we performed in vitro efferocytosis assays using CM-DiI red-labeled MΦ from myeloid-specific A1 knockout (M-A1^−/−^) mice and A1^f/f^ control mice. Confocal imaging revealed that A1^−/−^ MΦ exhibited impaired efferocytosis compared to control A1^f/f^ MΦ in efferocytosis assays in which engulfment of CFDA green-labeled R28 (Fig. 7A, B) or K562 (Fig. 7C, D) ACs by MΦ indicated efferocytosis events. Complementary flow cytometry studies confirmed that a higher percentage of CM-Dil red MΦ were positive for CFDA green-labeled ACs in MΦ populations from A1^f/f^ compared to M-A1^−/−^ mice (Fig. 7E, F).

### Inhibition of HDAC3 is neuroprotective in retinal IR injury

**Fig. 8:**
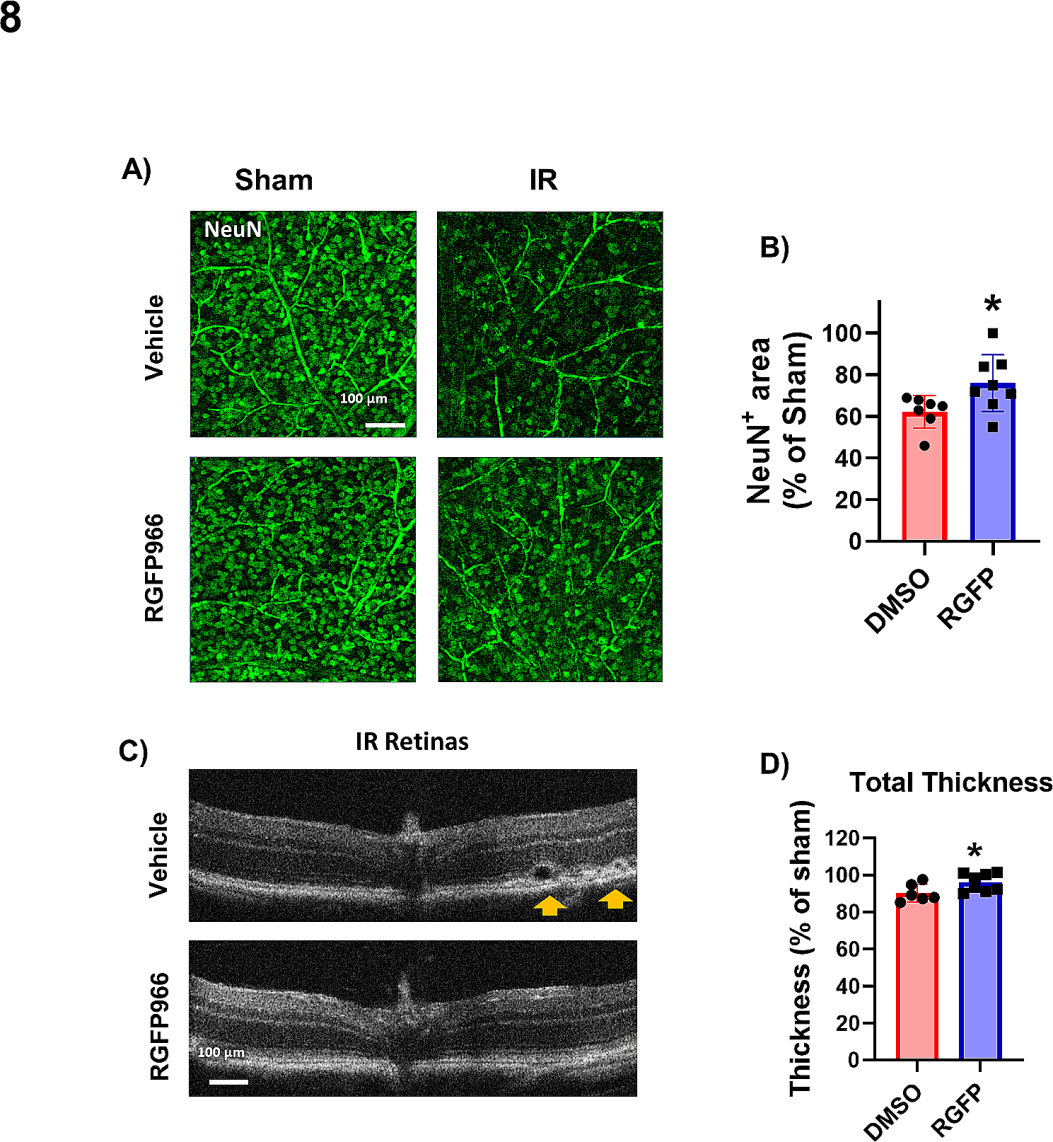
HDAC3 inhibition is neuroprotective and mitigates retinal thinning after IR injury. **A-B**) Retinal flat mounts from vehicle-treated WT mice show a marked loss of NeuN-labeled retinal neurons on day 7 after IR injury. After administration of the HDAC3 inhibitor RGFP966 (10 mg/kg i.p.) at 1 h after IR and on days 2, 4, and 6, retinal neurons were partially preserved at 7 days. RGFP996 did not reduce NeuN-positive cells in the retinas of the sham animals, *n* = 7–8. **C, D**) OCT images and quantification show preserved total retinal thickness on day 7 after IR injury in WT mice treated with RGFP966. Orange arrows indicate retinal detachment in the IR retinas of vehicle treated group that is absent in the RGFP996 treated group, *n* = 6–8, *p < 0.05

Based on our findings implicating HDAC3 in IR injury, we performed proof-of-principle in vivo studies to evaluate the therapeutic potential of HDAC3 inhibition. In these studies, WT mice were dosed with 10 mg/kg i.p. of the specific HDAC3 inhibitor RGFP966 or vehicle at 1 h after IR and every 48 h thereafter until day 7. The i.p. route of administration was selected because RGFP966 crosses the blood-retina barrier and no systemic toxicity has been reported using this dose [60]. Compared to vehicle (DMSO) treatment, administration of RGP966 conferred significant neuroprotection as revealed by the preservation of NeuN-positive neurons (Fig. 8A and B). OCT analysis also showed preserved retinal morphology and thickness in the RGFP966-treated mice (Fig. 8C and D). Of note, RGFP966 treatment had no effects on the contralateral retina thickness as compared to vehicle treatment (Supplementary Fig. S9). Collectively, HDAC3 inhibition showed similar neuroprotection to the myeloid-specific deletion of HDAC3.

## Discussion

Our study provides the first direct evidence to our knowledge that the HDAC3 isoform in myeloid cells suppresses efferocytosis and worsens ischemic retinopathy, and that reparative efferocytosis contributes to injury resolution in the inner retina. Compelling evidence that myeloid HDAC3 plays a detrimental role in retinal IR injury is provided by the observation of robust neurovascular protection in myeloid-specific HDAC3^−/−^ mice. This protection is mediated, at least in part, by enhanced efferocytosis in injured retinas of HDAC3^−/−^ mice that exhibited higher levels of myeloid cell association with apoptotic cells. Finally, pharmacological inhibition of HDAC3 protected against retinal IR injury, raising the possibility that HDAC3 may have value as a therapeutic target to mitigate retinal degeneration.

Initially, we considered different mediators of retinal IR injury including the HDAC gene family. Our interest in the HDAC family was based on evidence that HDAC pan-inhibitors limit neuronal damage after retinal IR injury [26, 27]. The HDAC3 isoform causes epigenetic modifications that promote the expression of MΦ inflammatory genes [30, 31]. Subsequently we observed a robust induction of HDAC3 in retinal myeloid cells in the mouse model of IR injury [5]. In the present study, we report colocalization of HDAC3 with the myeloid cell marker Iba1 in human retinas from control and DR, which is known to involve chronic ischemia. Interestingly, myeloid cells showed an amoeboid phenotype in the DR retinas denoting activation. Furthermore, publicly available data showed strong HDAC3 expression in human retinal microglia and vitreal MΦ. Building on these findings, we leveraged the use of myeloid-specific HDAC3^−/−^ mice to implicate HDAC3 in the neurovascular injury caused by retinal IR. Mice lacking HDAC3 in myeloid cells exhibited marked preservation of the inner retinal neurons as measured by NeuN labeling, which marks ganglion cells and other neuron cell types such as displaced amacrine cells [61, 62]. Mice lacking HDAC3 also showed preserved retinal thickness after IR injury. Retinal cell function evaluated by ERG was preserved in M-HDAC3^−/−^ mice. The losses of a- and b- wave amplitudes and OPs that are features of retinal injury and degeneration [1, 63, 64], and predict vision loss were less pronounced in retinas of M-HDAC3^−/−^ mice recovered for 14 days after IR insult. The partial preservation of the a-wave, b-wave, and OPs in the dark-adapted eye confirmed the collective protection of inner retina neurons as well as photoreceptors in these animals. In addition to its neuroprotective impact, deletion of myeloid HDAC3 conferred a robust retinal vascular protective effect as measured by reduced albumin extravasation and further confirmed using fluorescein angiography and Evans blue leakage. M-HDAC3^−/−^ mice retinas had fewer acellular retinal capillaries after IR injury as compared to retinas from HDAC3^f/f^ IR mice. Retinal permeability was measured at 48 h since previous research has shown that endothelial tight junction complexes exhibit significant disruption at this time point after IR [10]. Interestingly, While neurodegeneration occurs within the first week in this model, the death of endothelial cells does not happen until later, which is why we measured vascular degeneration at 14 days [6, 10]. This is in line with reports that retinal neurodegeneration precedes vascular degeneration [65, 66].

Our subsequent studies began to mechanistically clarify the contribution of HDAC3 to retinal IR injury. The retinal IR insult is known to induce a strong monocyte/MΦ cell response that also includes lesser microglial activation [10–12]. Accordingly, we recorded a 10-fold increase in myeloid leukocytes in the injured retina of floxed mice. In contrast, the retinas of M-HDAC3^−/−^ mice exhibited fewer microglia and myeloid leukocytes after IR injury as determined by flow cytometric analysis. This was confirmed by the reduced number of Iba1^+^ myeloid cells in M-HDAC3^−/−^ retina flat mounts after IR. However, the frequency of Ly6C^hi^ monocytes/MΦ remained the same, suggesting that HDAC3 deletion does not affect myeloid cell inflammatory response in retinal IR.

Our new finding that myeloid cells lacking HDAC3 exhibit enhanced efferocytosis after IR injury implies that HDAC3 suppresses the process of reparative efferocytosis in the retina. Two lines of evidence support this concept. First, Iba1^+^ myeloid cells lacking HDAC3 were observed to co-localize significantly more after IR injury with TUNEL^+^ apoptotic cells in retinal flat mount images. Second, HDAC3 deletion increased the number of phagocytic CD68^+^/PSVue^+^ microglia and myeloid leukocytes detected by flow cytometry in injured retinas. These collective results may indicate that despite the attenuation of myeloid cell proliferation in retinas of M-HDAC3^−/−^ mice after IR injury, the existing myeloid cell population can more efficiently engulf apoptotic cells to enact reparative efferocytosis without overactivation of the phagocytosis process. Indeed, our in vitro efferocytosis assays confirmed that MΦ lacking HDAC3 more efficiently clear apoptotic cells, pointing to HDAC3 as a culprit molecule that undermines retinal repair.

**Fig. 9:**
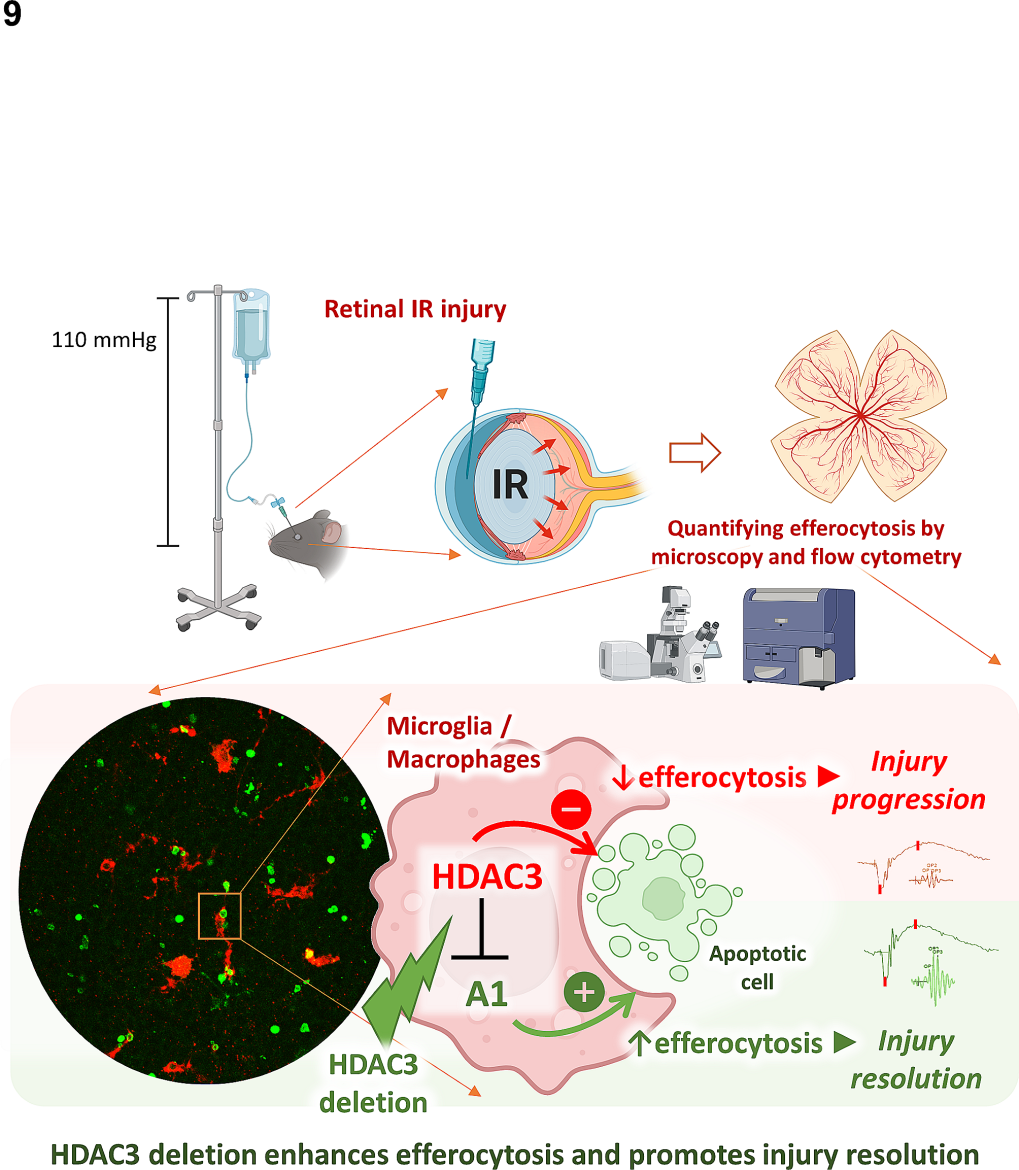
Schematic of the study findings. Myeloid HDAC3 suppresses efferocytosis and A1 expression. A1 promotes efferocytosis and plays a role in the enhanced efferocytosis of MΦ lacking HDAC3. HDAC3 may additionally impair efferocytosis by a mechanism unrelated to loss of A1

Finally, we explored whether HDAC3 deletion promotes efferocytosis by upregulating the known pro-efferocytic enzyme A1 [5], and whether an isoform-specific HDAC3 inhibitor confers neurovascular protection from IR injury. A1 transcript and protein were significantly higher in myeloid cells lacking HDAC3 and treated with apoptotic cells in vitro. Pharmacological inhibition of A1 blunted the enhanced efferocytosis conferred by HDAC3 deletion in MΦ. However, A1 inhibition had similar effects on control HDAC3^f/f^ MΦ. Thus, in addition to A1, there appear to be other unique and unidentified HDAC3 signaling pathways that modulate efferocytosis. The study findings are presented in Fig. 9, and ongoing studies in our lab are examining additional underlying mechanisms of HDAC3 inhibitory effect on efferocytosis using unbiased RNA-sequencing.

Regardless of the exact molecular pathways of protection, our final set of studies demonstrating that systemic administration of the HDAC3 inhibitor, RGFP966 to WT mice protects the retinal neurons and preserves retinal morphology after IR injury infers that HDAC3 may be a therapeutic target suitable for clinical intervention to preserve vision. Since RGFP966 readily penetrates the blood-retina barrier [60], systemic administration was selected over intravitreal injection to allow for multiple dosing. The dose and dosing interval were selected based on previous literature that showed maximal neuroprotection at 2 weeks post optic nerve crush with 10 mg/kg RGFP966, given i.p. every 3 days [60]. The same study showed that daily administration of RGFP966 at 10 mg/kg for 2 weeks did not result in abnormal histopathological effects or toxicity [60]. Furthermore, the 10 mg/Kg dose was shown to specifically inhibit HDAC3 with minimal inhibition of HDAC1 and HDAC2 [67]. While the plasma half-life of RGFP966 was reported to be relatively short (peaking within 30 min and declining over the next 2 h), our study and others show that it is protective in this short ‘pulsed’ dosing [68]. For human translation, localized HDAC3 inhibition via intravitreal administration could offer a more targeted approach compared to systemic administration, potentially avoiding systemic adverse effects.

In summary, we have identified the HDAC3 isoform expressed by myeloid cells as an important mediator of IR-induced retinal injury. HDAC3 appears to inhibit reparative efferocytosis in part through suppression of A1 expression. Further investigation into the mechanisms by which HDAC3 suppresses efferocytosis is warranted to facilitate the development of clinically effective HDAC3 inhibitors for the treatment of ischemic retinopathies.

### Electronic supplementary material

Below is the link to the electronic supplementary material.

Supplementary Material 1

## Data Availability

Data is provided within the manuscript or supplementary information files.
