# Supplementary material for: Deletion of myeloid HDAC3 promotes efferocytosis to ameliorate retinal ischemic injury
Source: J Neuroinflammation. 2024 Jul 12;21:170. doi: 10.1186/s12974-024-03159-8 (PMC11241909; doi:10.1186/s12974-024-03159-8)
Supplement: Supplementary file 1 — Supplementary Material 1 [file 12974_2024_3159_MOESM1_ESM.pdf]

Supplementary figures:

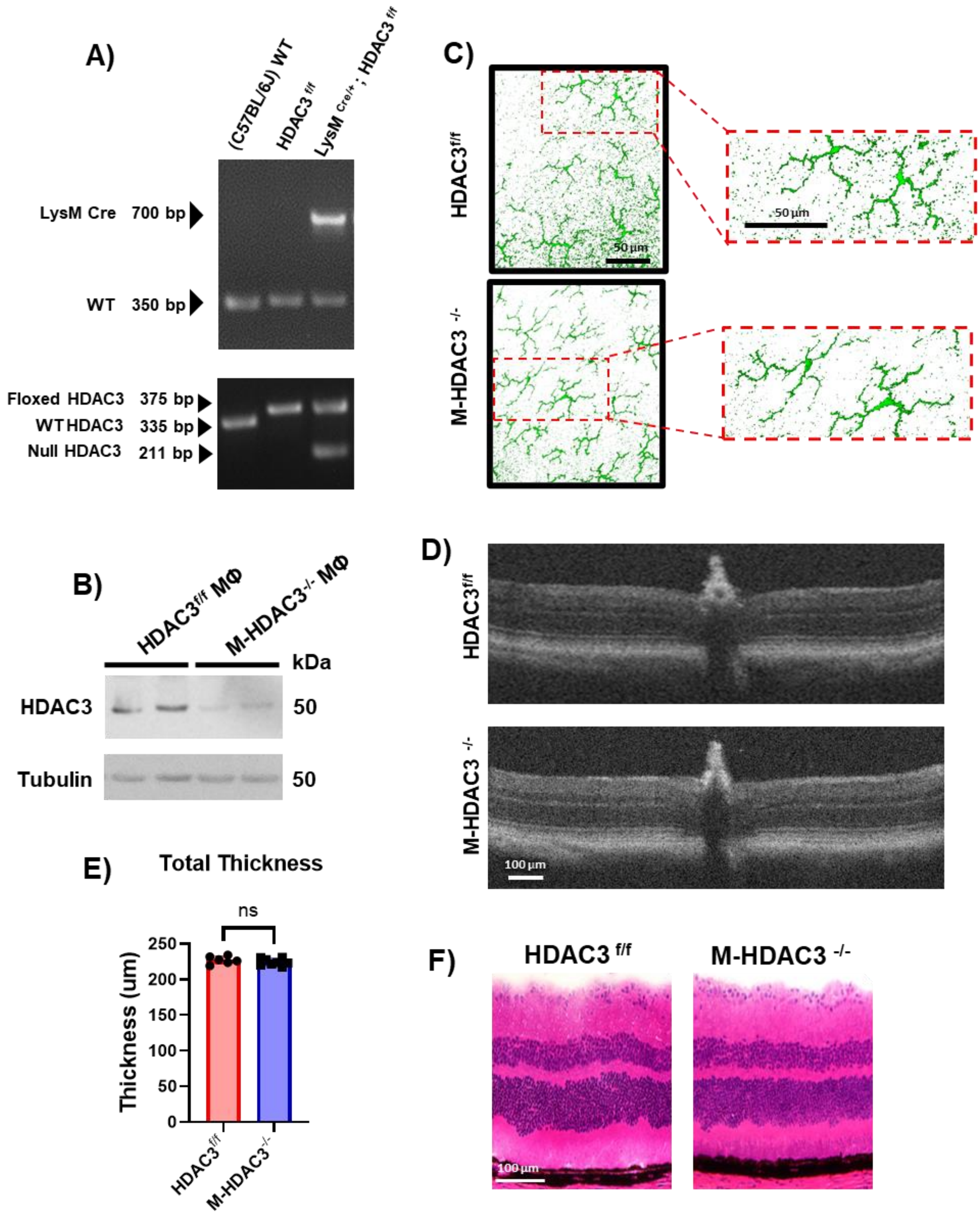

Figure S1. Characterization of myeloid-specific HDAC3 KO mice. A) PCR genotyping shows the LysM Cre band (700 bp) and the Null HDAC3 (211 bp) band demonstrating the successful generation of LysM$^{Cre/+}$; HDAC3$^{f/f}$ KO mice (M-HDAC3$^{-/-}$). **B)** HDAC3 deletion was confirmed by Western blot analysis of MΦ cell lysates

demonstrating the depletion of HDAC3 protein in M-HDAC3$^{-/-}$ MΦ. **C)** Immunofluorescent labeling of retinal flat mounts for Iba1 (microglia/ MΦ marker) reveals similar cell morphology between M-HDAC3$^{-/-}$ and HDAC3$^{f/f}$. **D, E)** OCT of M-HDAC3$^{-/-}$ and HDAC3$^{f/f}$ retinas in anesthetized mice and hematoxylin and eosin (H&E) staining of retinal sections (**F)** show similar retinal thickness and no morphological abnormalities in retinas of M-HDAC3$^{-/-}$ mice as compared to HDAC3$^{f/f}$ control mice, n=6-8, ns: not significant.

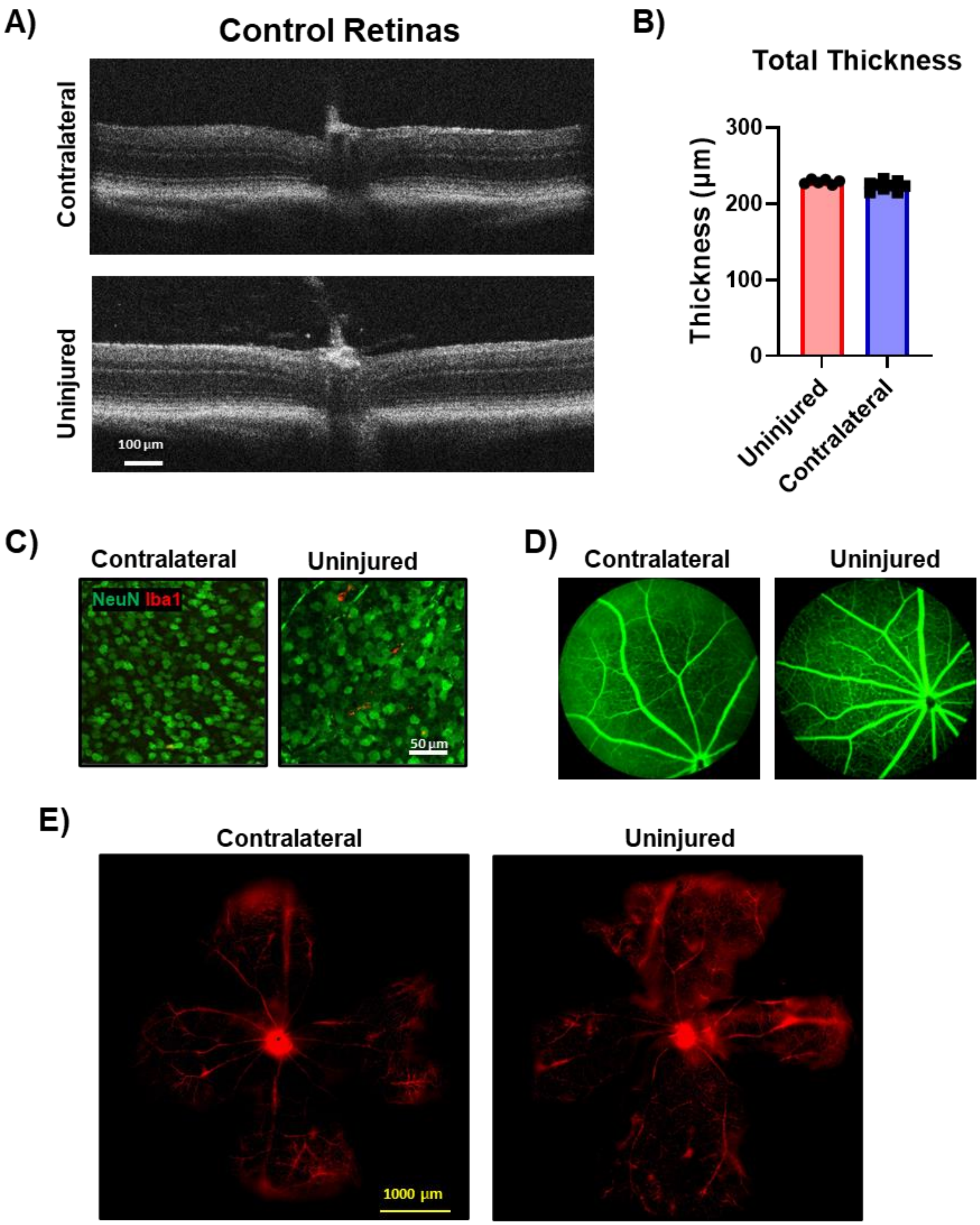

Figure S2. Characterization of retinas from the contralateral eye and uninjured control mice. We found no differences between the contralateral eye retinas and retinas from uninjured control mice as assessed using **A, B)** OCT, and **C)** NeuN/Iba1 labeling. **D)** fluorescein angiography, and **E)** Evans blue leakage showed preserved blood-retinal barrier integrity in both groups, n=6-9.

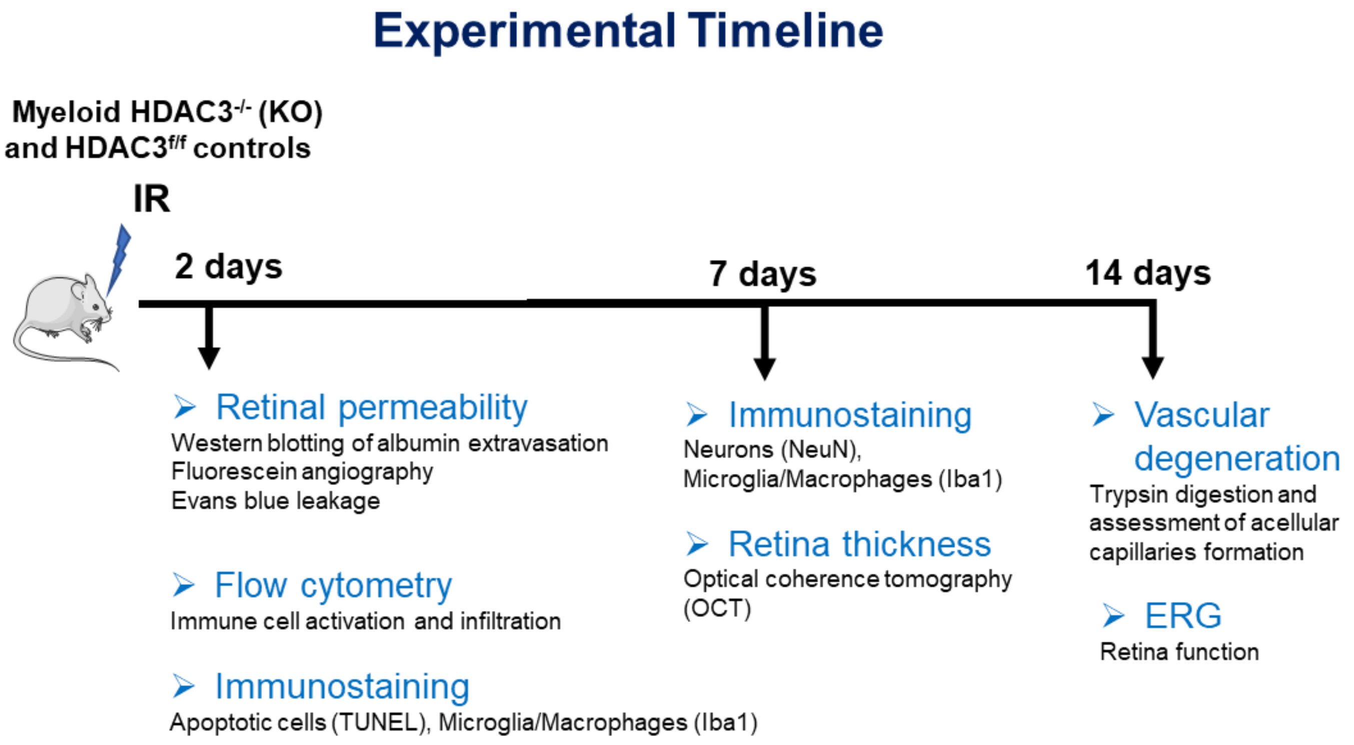

Figure S3. Schematic illustration of the *in vivo* experimental timeline.

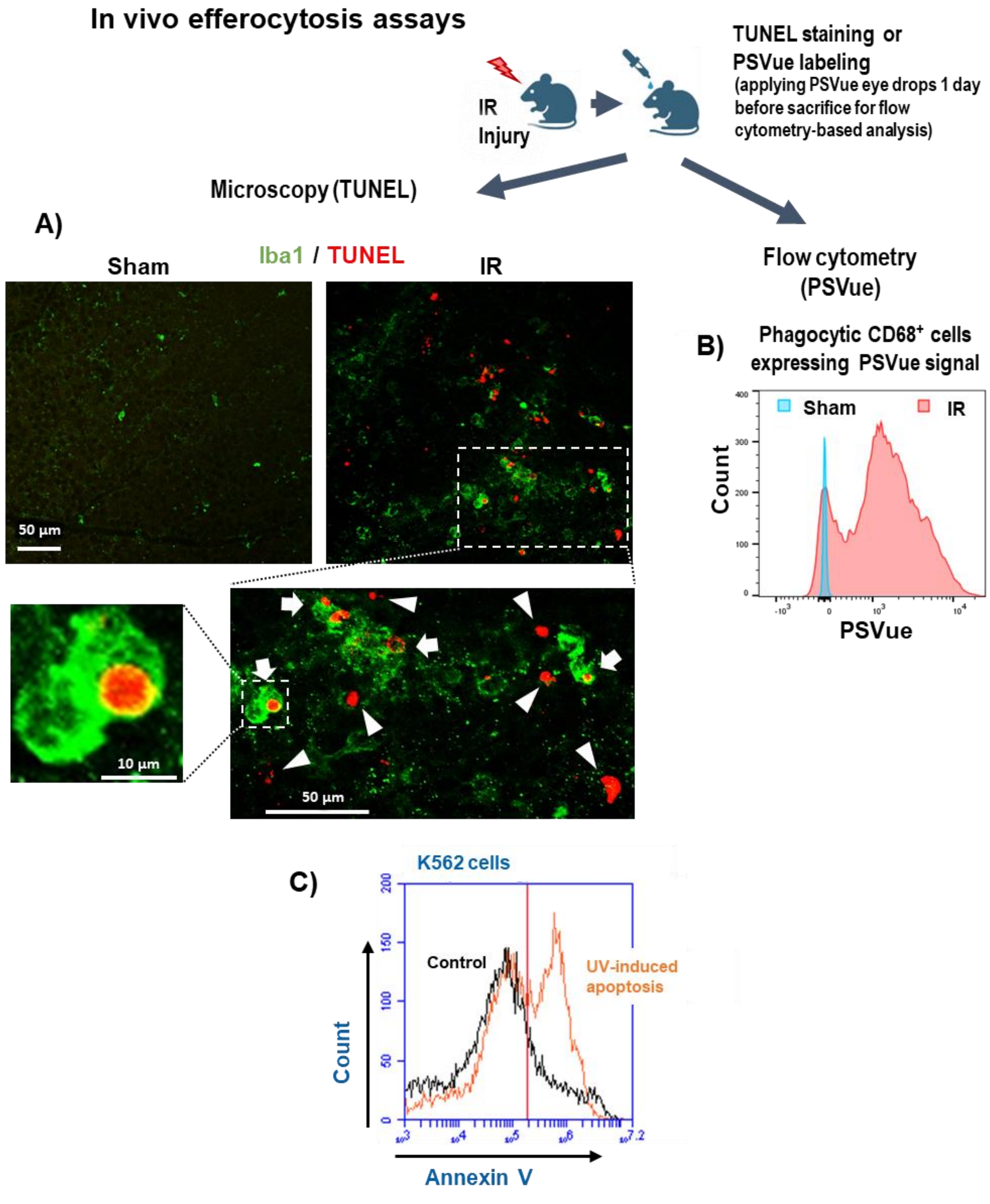

Figure S4. Efferocytosis assays. A schematic of *in vitro* efferocytosis assays conducted using TUNEL/Iba1 labeling of retina flat mounts at day 2 after IR **(A)** or flowcytometric analysis of $CD68^+$ phagocytic cells engaged in efferocytosis of $PSVue^+$ cells **(B)**. Arrows point to myeloid cells engulfing apoptotic cells whereas arrowheads point to unengulfed cells. **C)** Confirmation of apoptosis induction in K562 cells by UV-B irradiation for 15 minutes using annexin V labeling.

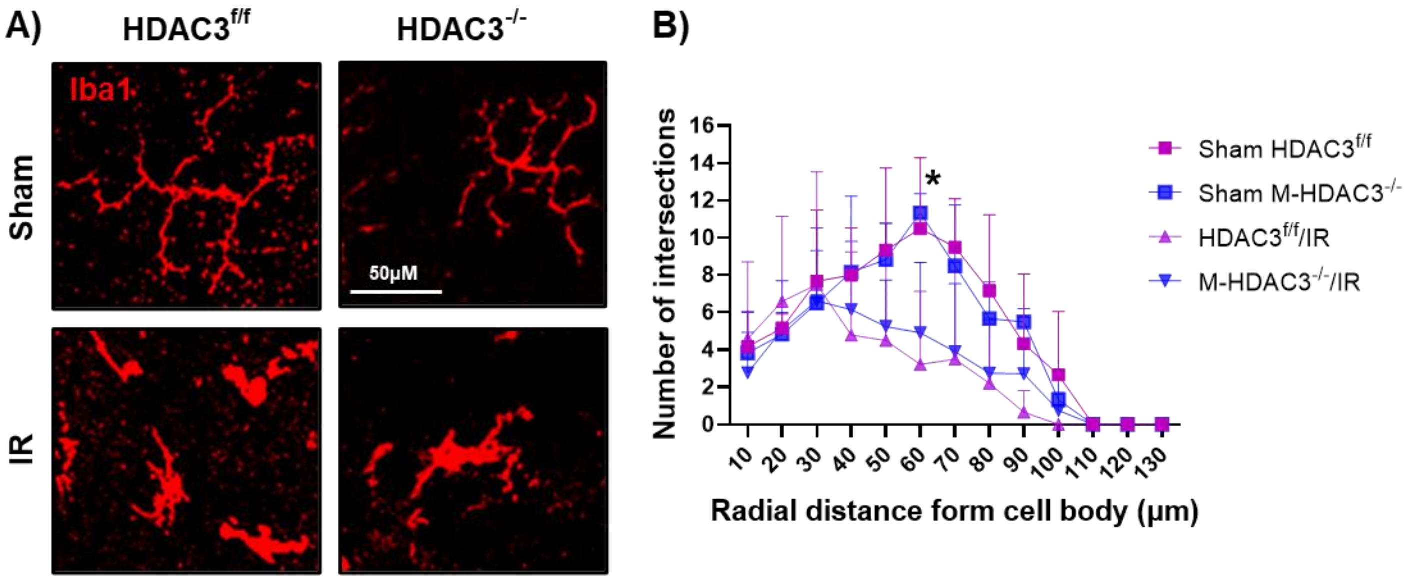

Figure S5. Morphological analysis of retinal myeloid cells. A) Representative confocal microscopy images of retinal flat mounts stained with Iba1 (green) from HDAC3$^{f/f}$ and M-HDAC3$^{-/-}$ Mice at 2 days post-IR injury. **B)** Sholl analysis of retinal Iba^{+} myeloid cells demonstrating decreased processes complexity after IR injury. This reduction is indicated by fewer intersections on concentric circles spaced 10 μm apart from the cell body. ANOVA analysis showed statistical significance between IR and sham groups (*p<0.05) but no statistical differences within the IR or sham groups, n= 3-4.

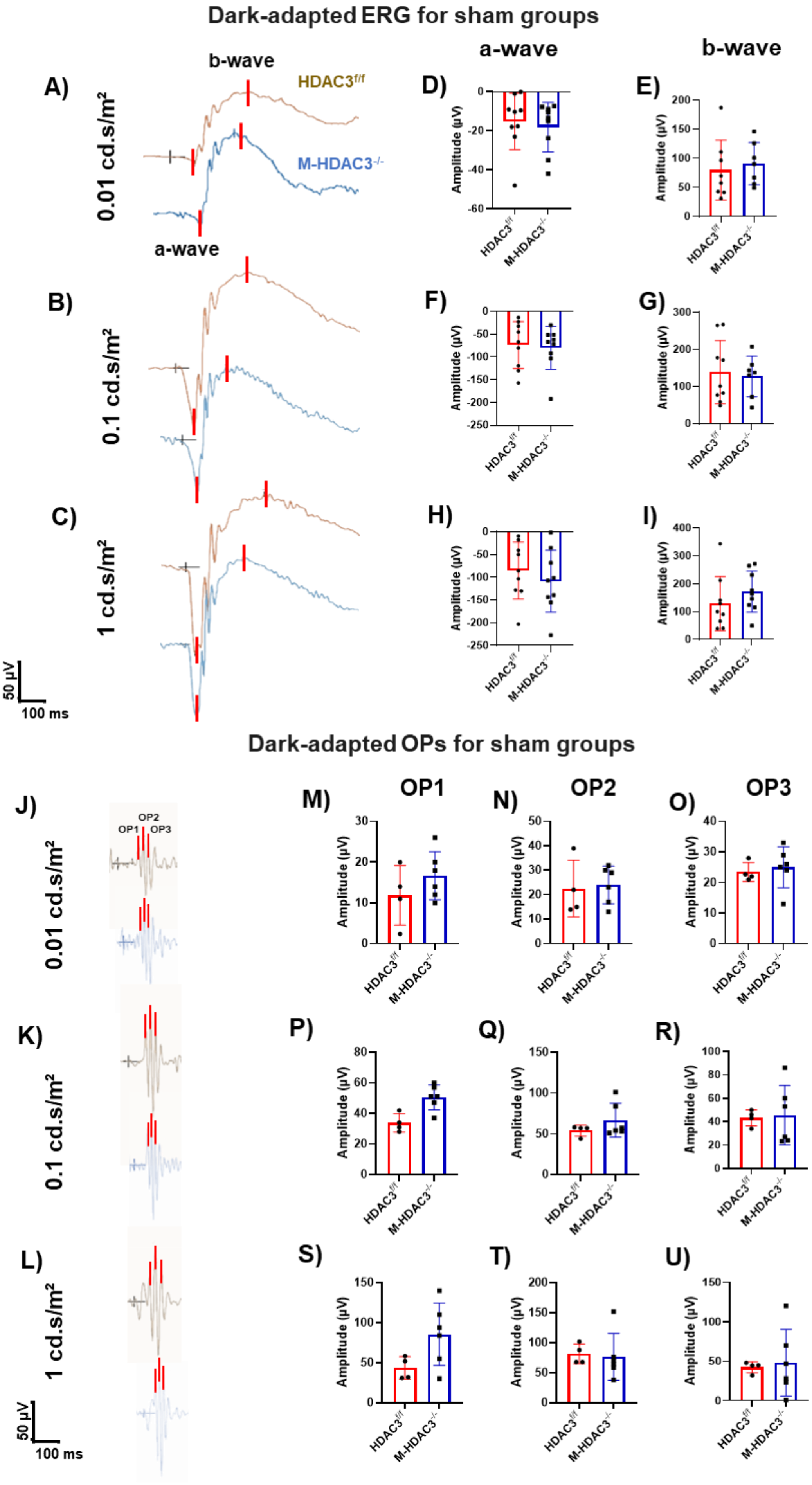

Dark-adapted ERG for sham groups
a-wave
b-wave
A)
B)
C)
D)
E)
F)
G)
H)
I)
0.01 cd.s/m²
0.1 cd.s/m²
1 cd.s/m²
HDAC3f/f
M-HDAC3-/-
Amplitude (µV)
50 µV
100 ms
Dark-adapted OPs for sham groups
OP1
OP2
OP3
J)
K)
L)
M)
N)
O)
P)
Q)
R)
S)
T)
U)

Figure S6. Characterization of dark-adapted ERG and OPs in Sham M-HDAC3$^{-/-}$ and HDAC3$^{f/f}$ mice. A-C) Representative ERG waveforms of un-injured (sham) M-HDAC3-/- and HDAC3f/f mice at 0.01, 0.1, and 1 cd.s/m^2 light intensities. **D-I)** Quantification and comparison of amplitudes show no change in a-wave and b-wave responses in M-HDAC3$^{-/-}$ retinas as compared to HDAC3$^{f/f}$. **J-U)** Similarly, oscillatory potentials (OPs) graphs and quantifications at various light intestines show no change between the M-HDAC3$^{-/-}$ and HDAC3$^{f/f}$ retinas, n=4-6.

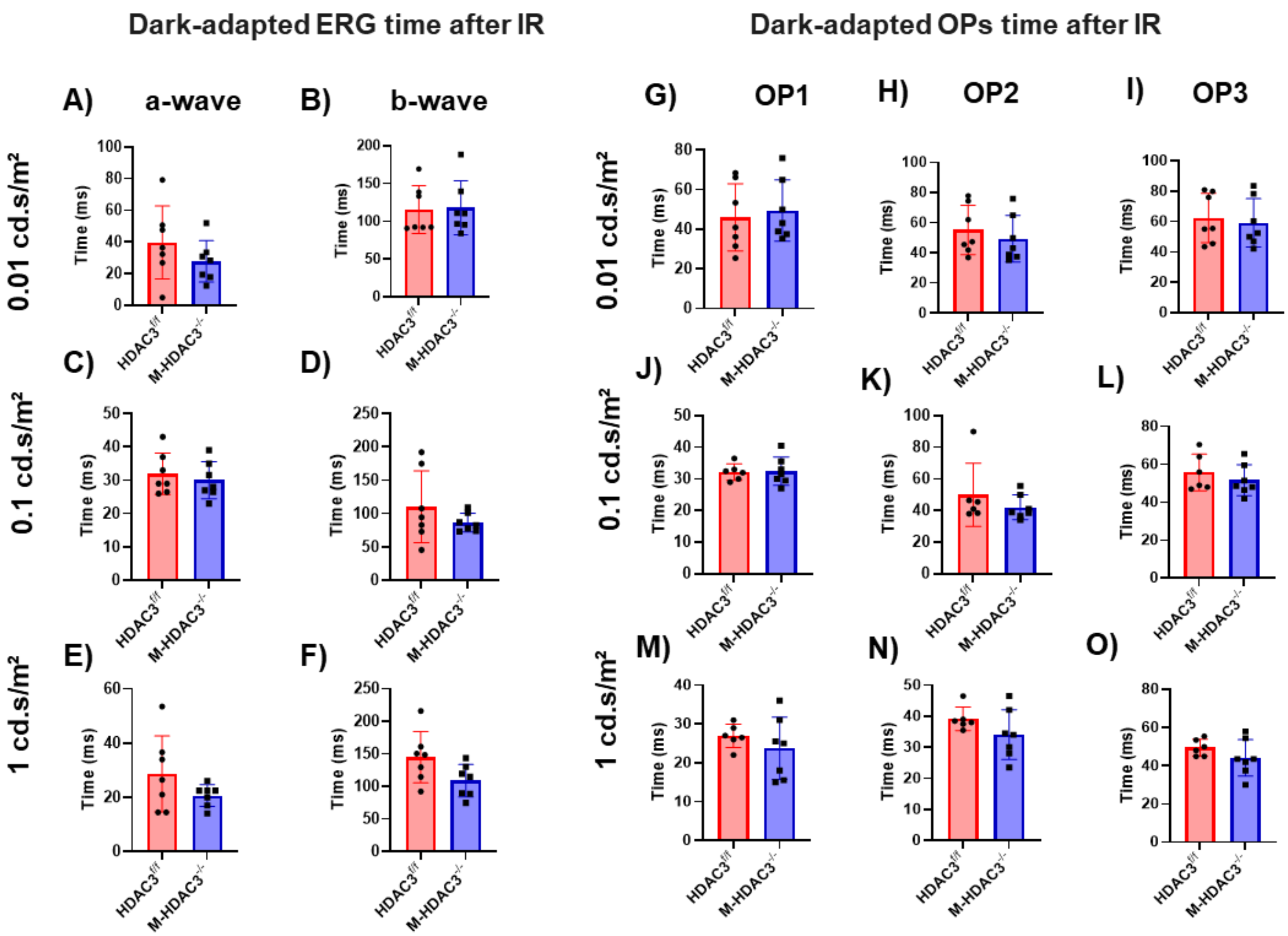

Figure S7. Characterization of dark-adapted ERG and OPs time in IR injured M-HDAC3$^{-/-}$ and HDAC3$^{f/f}$ mice. A-F) Quantification and comparison of amplitudes at 0.01, 0.1, and 1 cd.s/m2 light intensities show no change in a-wave and b-wave responses in IR injured M-HDAC3$^{-/-}$ retinas as compared to injured HDAC3$^{f/f}$. G-O) Similarly, oscillatory potentials (OPs) graphs and quantifications at various light intestines show no change between the inured M-HDAC3$^{-/-}$ and HDAC3$^{f/f}$ retinas, n=7 per group.

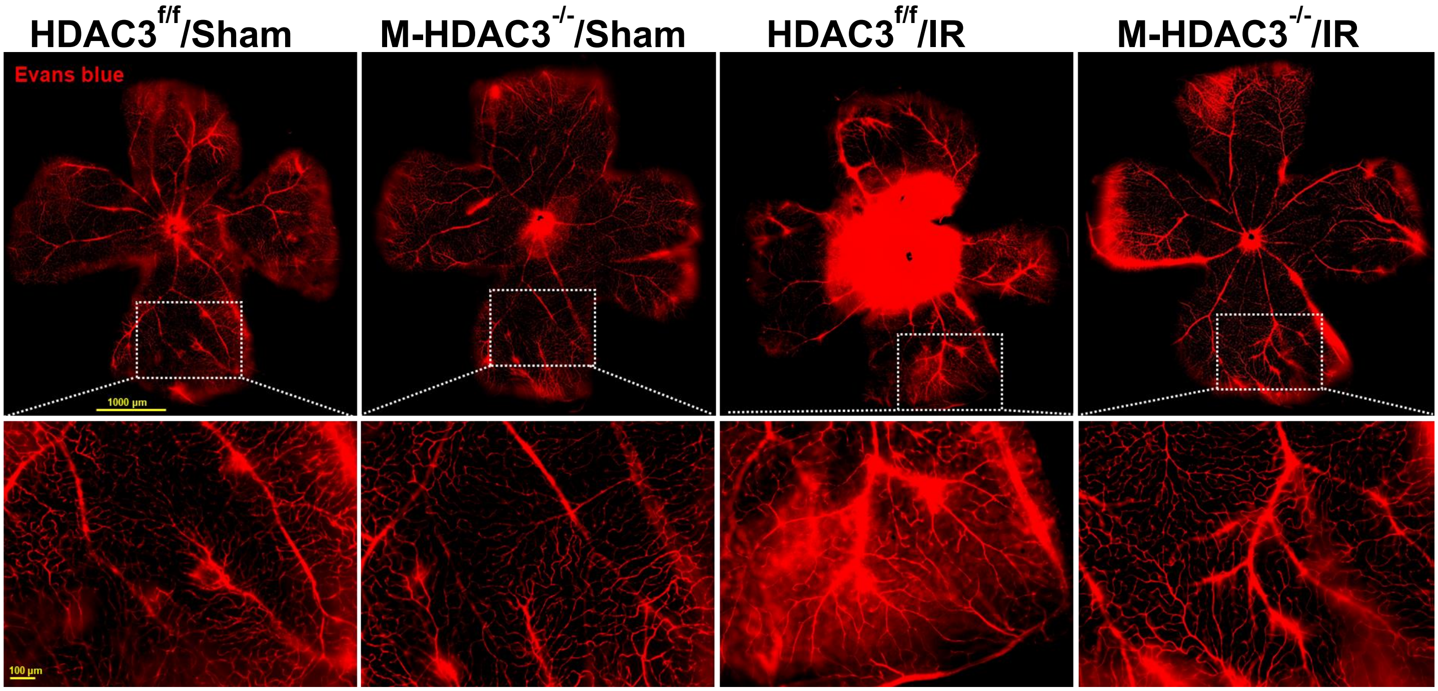

Figure S8. HDAC3 deletion mitigates Evans blue vascular leakage in the mouse retina after IR injury. Representative flat mount images collected 48 h following IR injury demonstrating vascular leaking of Evans blue dye (red color) after IR. M-HDAC3$^{-/-}$ mice showed reduced Evans blue leakage compared to the M-HDAC3$^{f/f}$ mice retinas, n=4-5 per group.

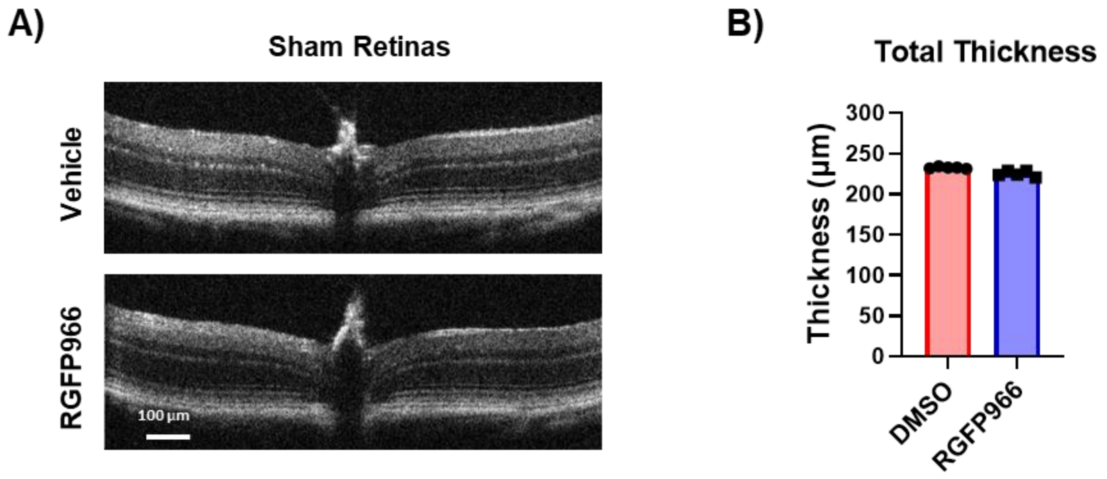

Figure S9. Measurement of retinal thickness in sham retinas treated with the HADC3 inhibitor, RGFP966, or vehicle (DMSO). A, B) Representative OCT images and quantification of retinal thickness showed no statistical differences between sham retinas treated with RGFP966 or vehicle, n=5 per group.

Blots for figure 3 A)

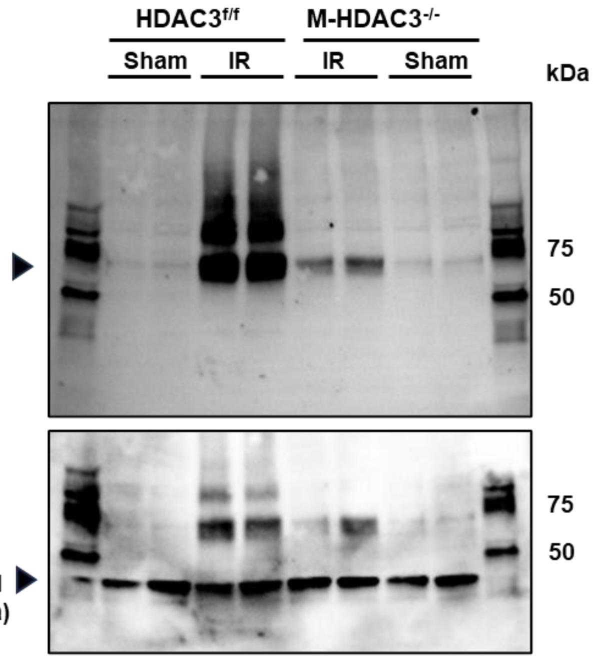

Blots for figure 6 C)

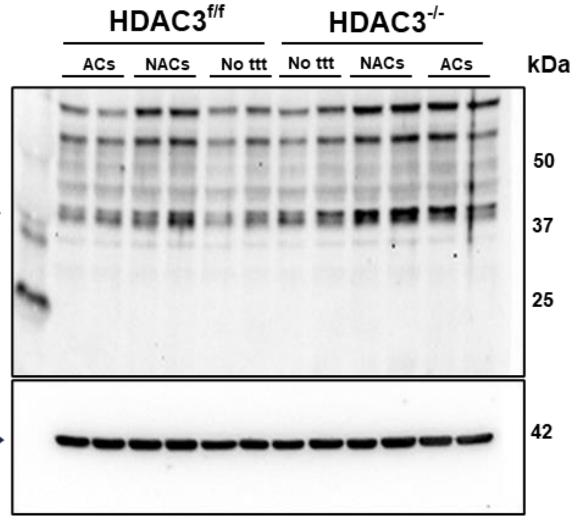

Blots for figure S1 B)

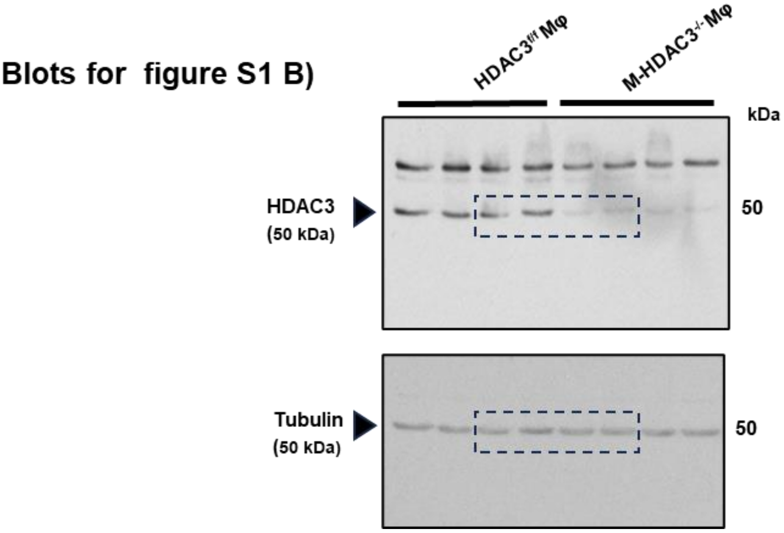

Figure S10. Full, uncropped images of the representative Western blots included in the manuscript.
